# Practical Recommendations for Exercise Training in Patients with Long COVID with or without Post-exertional Malaise: A Best Practice Proposal

**DOI:** 10.1186/s40798-024-00695-8

**Published:** 2024-04-24

**Authors:** Rainer Gloeckl, Ralf H. Zwick, Ulrich Fürlinger, Tessa Schneeberger, Daniela Leitl, Inga Jarosch, Uta Behrends, Carmen Scheibenbogen, Andreas Rembert Koczulla

**Affiliations:** 1 Department of Pulmonary Rehabilitation, Philipps-University of Marburg, Marburg, Germany; 2 Institute for Pulmonary Rehabilitation Research, Schoen Klinik Berchtesgadener Land, Schoenau am Koenigssee, Germany; 3 Therme Wien Med, Ludwig Boltzmann Institute for Rehabilitation Research, Vienna, Austria; 4 Childrens’ Hospital, School of Medicine, Technical University of Munich, Munich, Germany; 5 German Center for Infection Research (DZIF), Berlin, Germany; 6 Institute of Medical Immunology, Charité - Universitätsmedizin Berlin, corporate member of Freie Universität Berlin and Humboldt Universität Zu Berlin, Berlin, Germany; 7 Teaching Hospital, Paracelsus Medical University Salzburg, Salzburg, Austria

**Keywords:** Rehabilitation, Pulmonary rehabilitation, Post-COVID, COVID-19, SARS-CoV-2, Fatigue, Post exertional symptom exacerbation, PEM, PESE

## Abstract

People with long COVID may suffer from a wide range of ongoing symptoms including fatigue, exertional dyspnea, reduced exercise performance, and others. In particular, impaired exercise performance is a condition that can be recovered in many people through an individualized physical exercise training program. However, clinical experience has shown that the presence of post-exertional malaise (PEM) is a significant barrier to physical exercise training in people with long COVID. Currently, there is no guideline or consensus available on how to apply exercise training in this cohort. Therefore, we conducted a literature review in the PubMed library using the following search terms: “COVID”, “post-COVID”, “long COVID” and “exercise” searching for studies from January 2020 to January 2024. Data from 46 trials were included. Exercise training regimes were very heterogeneous and none of these studies reported on the management of PEM in the context of an exercise training program. Based on the feedback from an additional survey that was answered by 14 international experts in the field of exercise training in long COVID, combined with the authors´ own extensive practical experience, a best practice proposal for exercise training recommendations has been developed. This proposal differentiates exercise procedures according to the presence of no, mild/moderate or severe PEM in people with long COVID. These recommendations may guide allied healthcare professionals worldwide in initiating and adjusting exercise training programs for people with long COVID, stratified according to the presence and severity of PEM.

**Supplementary Information:**

The online version contains supplementary material available at 10.1186/s40798-024-00695-8.

## Key Points

- Exercise training is considered as a crucial component of long COVID rehabilitation programs, but it may be complicated by the presence of post-exertional malaise.
- Currently, there is no consensus on the optimal training strategies for individuals with long COVID, with or without post-exertional malaise (PEM).
- We propose a best practice proposal for exercise training recommendations that distinguishes between exercise procedures based on the presence of no, mild/moderate or severe PEM in individuals with long COVID.
- Our best-practice proposal, which is based on scientific literature and extensive expert experience, can guide healthcare professionals in initiating and adjusting exercise training programs for individuals with long COVID, stratified according to the presence and severity of PEM.

**Supplementary Information:**

The online version contains supplementary material available at 10.1186/s40798-024-00695-8.

## Background

According to the current NICE guideline, long COVID is a term used to describe signs and symptoms that persist or develop for more than four weeks after the acute phase of COVID-19 [1]. People with long COVID may experience a wide range of ongoing symptoms including fatigue, exertional dyspnea, psycho-neurological impairments, pain, reduced exercise performance, and others [2]. In particular, impaired exercise performance is a condition that can be recovered by an individualized physical exercise training program. Recent systematic reviews have shown that physical rehabilitation interventions are feasible, safe, and beneficial in people with long COVID by improving various physical, clinical, and psychological relevant outcomes [3]. In addition, long COVID-related symptoms such as exertional dyspnea or fatigue have been shown to improve following exercise training interventions [4]. However, a recent Cochrane review concluded that the available evidence has several methodological limitations that prevent the formulation of robust suggestions for exercise practice [5]. In particular, the presence of post-exertional malaise (PEM), a worsening of symptoms following physical, cognitive, or emotional activity that typically intensifies 12–48 h after an activity and lasts for days or even weeks [6], is a significant barrier to physical exercise training in long COVID. Up to now, there are no consistent exercise training recommendations for people with long COVID available. Therefore, we aimed to develop practical exercise training recommendations for individuals with long COVID, depending on the presence and severity of PEM.

## Methodological Aspects

**Fig. 1:**
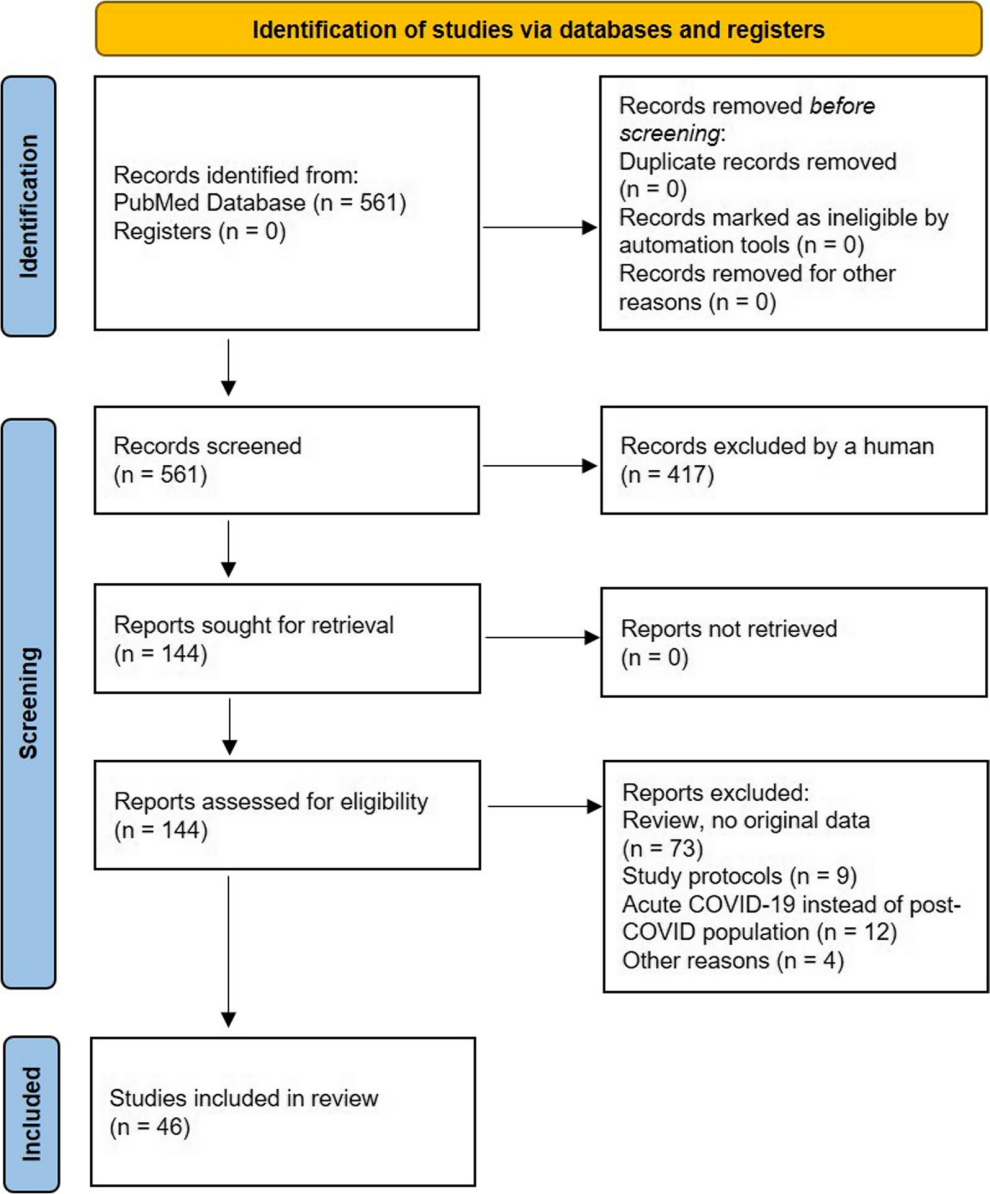
Literature search flow chart

Information on exercise training procedures in people with long COVID was derived from three different sources. First, a systematic literature search was performed in July 2023 and was updated in January 2024. The electronic search was carried out in the PubMed library using a search period from January 2020 to January 2024 and the following keywords: “COVID”, “Post-COVID”, “long COVID” and “exercise”. We included trials that used any kind of physical exercise training program in people with persistent symptoms related to COVID. There were no restrictions on study methodology (e.g. randomized, observational, retrospective etc.). As part of the study selection process, two independent researchers (R.G. and D.L.) screened the titles and the abstracts of the articles, reviewed the full text of all articles that met the inclusion criteria in the initial screening, and extracted data from the eligible studies (Fig. 1). Second, we developed a 48-question online survey on how exercise training is initiated and adapted in people with long COVID (Additional file 1). This survey was sent to international long COVID experts who have published studies on exercise training in people with long COVID to gather additional practical experiences beyond the reported details in publications. Thirdly, practical experiences from the four authors´ expert centers [7–9] after treating more than 3500 people with long COVID so far were also taken into consideration to create practical exercise training recommendations.

## Findings

**Fig. 2:**
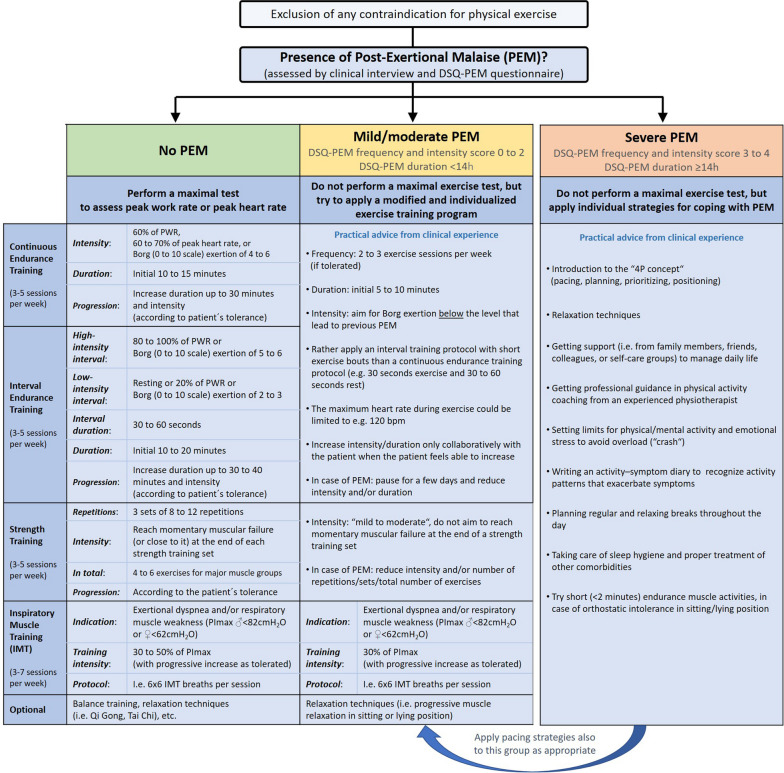
A best-practice proposal for exercise training recommendations in patients with long COVID (bpm = beats per minute, DSQ-PEM = DePaul Symptom Questionnaire-Post-Exertional Malaise, IMT = inspiratory muscle training, PImax = maximal inspiratory pressure, PWR = peak work rate)

The literature search revealed 46 original studies that investigated exercise training programs in individuals with long COVID (for extracted information on exercise training procedures see Table 1). Most studies performed 3–5 exercise training sessions per week for a period of 3–12 weeks. However, approaches to exercise prescription in the scientific literature were very heterogeneous. To determine training intensity during endurance training, various methods were employed. Ten studies used a percentage of peak heart rate, ranging from 40 to 85%. Eight studies used Borg exertion scores, ranging from 3 to 6 on the 0–10 point scale. Seven studies applied a percentage of peak work rate, ranging from 20 to 70%. Four studies used a percentage of heart rate reserve, ranging from 30 to 70%. Ten studies did not report how they determined intensity and seven studies did not apply endurance training (Table 1). Twenty of the 46 trials (43%) reported on the safety of exercise training in people with long COVID (without mentioning PEM). None of these trials documented any exercise-related adverse events. However, 57% of the included trials did not report the prevalence of exercise-related adverse events. Additionally, 14 experts (across 8 countries) also completed the survey. The responses from these experts were also very heterogeneous (e.g. endurance training intensity ranged from 30 to 70% of peak heart rate or 30–60% of peak work rate). Based on the literature review, the expert survey, and the authors’ own extensive experience, a proposal for practical exercise recommendations for people with long COVID stratified by the presence, frequency, severity, and duration of PEM was developed (Fig. 2).

**Table 1:** Overview of exercise prescriptions in studies investigating exercise training programs in individuals with long COVID

Study	Reference no.	Inclusion of people with long COVID and fatigue symptoms?	Assessing for PEM?	Endurance training modalities				Strength training modalities		IMT modalities		Training-related serious adverse events
				Intensity	Continuous or interval training mode	Interval modality	Total duration [min]	Intensity	Sets and repetitions	Intensity	Duration	
Liu et al. 2020	[10]	Not reported	Not reported	NA				NA		60% PImax	3 × 10 reps	Not reported
Gloeckl et al. 2021	[7]	Yes	Not reported	60–70% PWR	CT	–	10–20 min	15–20 RM	3 × 15–20 reps	NA		No adverse events
Daynes et al. 2021	[11]	Yes	Not reported	Not reported		–	Not reported	Not reported		NA		No adverse events
Abodonya et al. 2021	[12]	Not reported	Not reported	NA				NA		50% of MIP	6 × 5 min	Not reported
Dalbosco-Salas et al. 2021	[13]	Yes	Not reported	Borg 3–6 (0–10 scale)	CT	–	20–30 min	NA		NA		Not reported
Martin et al. 2021	[14]	Yes	Not reported	Borg 6 (0–10 scale)	CT	–	30 min	8–12 RM	3 × 8–12 reps	NA		No adverse events
Nambi et al. 2021	[15]	Not reported	Not reported	40–60% or 60–80% of peak HR	CT	–	30 min	10RM	3 × 10 reps	NA		Not reported
Stavrou et al. 2021	[16]	Yes	Not reported	75% of peak HR	CT	–	50 min	Not reported	2 × 12 reps	NA		Not reported
Mohamed et al. 2021	[17]	Yes	Not reported	60–75% of peak HR	CT	–	30 min	NA		NA		Not reported
Betschart et al. 2021	[18]	Yes	Not reported	20–30% PWR	CT	–	30 min	50–85% of 1RM		3 × 10–12 reps	NA	No adverse events
Hayden et al. 2021	[19]	Yes	Not reported	Borg 4–6 (0–10 scale)	CT	–	30–60 min	12 RM	3 × 12 reps	NA		Not reported
Spielmanns et al. 2021	[20]	Not reported	Not reported	55–70% of peak HR	IT	30–60 s	10–30 min	12 RM	3 × 12 reps	NA		Not reported
Udina et al. 2021	[21]	Not reported	Not reported	Borg 3–5 (0–10 scale)	CT	–	15 min	30–80% of 1RM	2 × 10 reps	NA		Not reported
Zampogna et al. 2021	[22]	Not reported	Not reported	Borg 4–5 (0–10 scale)	CT	–	20–30 min	NA		NA		Not reported
Bouteleux et al. 2021	[23]	Yes	Not reported	Not reported	–	–	–	Not reported	–	NA		Not reported
Albu et al. 2021	[24]	Yes	Not reported	Not reported	CT	–	20–30 min	12 RM	3 × 12 reps	30% PImax	3 × 3 min	Not reported
Al Chikhanie et al. 2021	[25]	Yes	Not reported	Not reported	–	–	–	Not reported	–	NA		Not reported
Besnier et al. 2022	[26]	Yes	Not reported	first ventilatory threshold (VT1)	CT	–	30 min	40% of 1RM	3 × 10 reps	Not reported	3 × 10 reps	Not reported
Jimeno-Almazan et al. 2022	[27]	Yes	Not reported	70–80% of HRR vs. 55–65% of HRR	IT	4–6 × 3–5 min	30 min	50% of 1RM	3 × 8 reps	NA		No adverse events
Li et al. 2022	[28]	Not reported	Not reported	40–60% of HRR	CT	–	45–60 min	NA		NA		No adverse events
Capin et al. 2022	[29]	Not reported	Not reported	Not reported	IT	10 s to 5 min	Not reported	8 RM	1 × 8 reps	NA		No adverse events
McNarry et al. 2022	[30]	Not reported	Not reported	NA	Not reported	–	NA	NA		> 80% of PImax	6 × 6 reps	Not reported
Nopp et al. 2022	[8]	Yes	Not reported	30–70% PWR	IT	60 Sec	20 min	8–15RM	3 × 8–15 reps	80% PImax	1 × 20 reps	No adverse events
Contreras-Briceno et al. 2022	[31]	Yes	Not reported	30–60% of HRR	CT	–	40–60 min	NA		NA		Not reported
Hockele et al. 2022	[32]	Not reported	Not reported	Not reported	CT	–	20 min	„Light to intense “	3 × 10 reps	30% PImax	–	Not reported
Teixeira do Amaral et al. 2022	[33]	Not reported	Not reported	Borg 11–13 (6–20 scale)	CT	–	30 min	Borg 15–17 (6–20 scale)	3 × 15–20 reps	NA		No adverse events
Palau et al. 2022	[34]	Not reported	Not reported	NA				NA		30% PImax	20 min	No adverse events
Estebanez-Pérez et al. 2022	[35]	Yes	Not reported	Not reported	CT	–	20–30 min	Not reported	3 × 8–12 reps	NA		No adverse events
Rutkowski et al. 2022	[36]	Yes	Not reported	60–80% of submaximal HR	CT	–	30 min	Not reported		NA		Not reported
Corna et al. 2022	[37]	Not reported	Not reported	55–85% of peak HR	CT	–	20 min	NA		NA		No adverse events
Vitacca et al. 2022	[38]	Yes	Not reported	70%PWR or 100% / 40% PWR	CT and IT	–	–	NA		NA		No adverse events
Asimakos et al. 2023	[39]	Yes	Not reported	50% PWR	IT	30 Sec	30 min	60–70% of 1RM	3 × 10 reps	NA		No adverse events
Ostrowska et al. 2023	[40]	Yes	Not reported	Not reported	–	–	Not reported	–	–	NA		Not reported
Jimeno-Almazan et al. 2023	[41]	Yes	Not reported	70–80% of HRR vs. 55–65% of HRR	CT and IT	–	–	–	–	NA		No adverse events
Spielmanns et al. 2023	[42]	Not reported	Not reported	55–70% of peak HR	IT	30–60 s	10–30 min	12 RM	3 × 12 reps	NA		Not reported
Colas et al. 2023	[43]	Not reported	PEM was exclusion criteria	Not reported			90 min	Not reported	Not reported	NA		Not reported
Alsharidah et al. 2023	[44]	Not reported	Not reported	60–80% of peak HR	CT		20–30 min	10 RM	3 × 10 reps	NA		No adverse events
Ghasemi et al. 2023	[45]	Not reported	Not reported	NA			NA	65–75% of 1RM	12–15 reps	NA		Not reported
Minko et al. 2023	[46]	Not reported	Not reported	Not reported	CT and IT	Not reported	90 min	70–85% of 1RM	8–12 reps	NA		Not reported
Espinoza-Bravo et al. 2023	[47]	Yes	Not reported	Borg dyspnea score 4 (0–10 scale)	CT		25–45 min	Not reported	2–3 × 10 reps	NA		No adverse events
Mooren et al. 2023	[48]	Yes	Not reported	50% or 60%/30% PWR	CT and IT	50–100 s	18 min	NA	NA	NA		Not reported
Del Corral et al. 2023	[49]	Yes	Not reported	NA			NA	NA	NA	20–80% PImax	6–10 reps	No adverse events
Rodriguez-Blanco et al. 2023	[50]	Yes	Not reported	NA			NA	Not reported	12 reps	NA		Not reported
Romanet et al. 2023	[51]	Yes	Not reported	60–70% of PWR or Borg dyspnea 4–6 (0–10 scale)	CT		15–60 min	Training until muscle fatigue	4 × 6–12 reps	NA		Not reported
Kerling et al. 2024	[52]	Yes	Not reported	60–75% of peak HR	CT			Not reported	Not reported	NA		Not reported
Pietranis et al. 2024	[53]	Yes	Not reported	45–55% of peak HR or 70–80% of peak HR	CT and IT	120–240 s	15–45 min	Not reported	8–12 reps	45–80% of PImax	6 × 6 reps	No adverse events

6MWT—6-min walk test, CT—continuous endurance training, HR—heart rate, HRR—heart rate reserve, IMT—inspiratory muscle training, IT—interval endurance training, MIP—maximum inspiratory pressure, NA—not applied, PImax—maximal inspiratory pressure, PEM—post-exertional malaise, PWR—peak work rate, Ref—reference, RM—repetition maximum, reps—repetitions, sec—seconds

Before starting an exercise program, a cardiac and pulmonary assessment should be performed to exclude potential contraindications to exercise training. Another crucial aspect before prescribing exercise training is to assess for PEM (by clinical interview and if suspected by using the DePaul Symptom Questionnaire-Post-Exertional Malaise, DSQ-PEM) [54]. PEM is also the cardinal symptom of myalgic encephalomyelitis/chronic fatigue syndrome (ME/CFS) and therefore in long COVID cases with PEM, diagnostic ME/CFS criteria (Canadian Consensus or IOM criteria) should be checked [55, 56]. If there are no signs of PEM, a “conventional” exercise training program that combines moderate to intense endurance and strength training may be used. However, if people develop PEM after daily physical activity an individual activity management strategy known as pacing or energy envelope maintenance [57] should be applied (Fig. 2).

## Discussion

We have presented best practice recommendations for exercise training in people with long COVID, based on a mixture of scientific literature, an international expert survey and our own practical experience. We believe that differentiating whether people with long COVID have a history of PEM and/or develop PEM after exercise is an important consideration concerning the adaptation of exercise training programs. If subjects with long COVID do not develop PEM, a fairly regular fitness training program of combined endurance and strength training could be applied as in healthy untrained individuals. If people develop mild to moderate PEM, a modified exercise training program might be used (depending on individual tolerance). For people with long COVID and severe PEM, the focus should be on pacing strategies similar to those used in patients with ME/CFS.

In January 2023, the World Health Organization (WHO) suggested that people with long COVID and significant impact on everyday functioning should be referred to rehabilitation services [58, 59]. There was also a recommendation that the presence of PEM will require interventions to be modified without mentioning further details [58]. The WHO, as well as the Cochrane Institute, concluded that there is currently no direct evidence of the effectiveness of rehabilitation in the subgroup of people with long COVID and PEM [58, 60]. Our literature review supports these statements since none of the 46 studies specifically reported on PEM (Table 1). However, 12 out of 14 survey participants stated that long COVID individuals should be screened for PEM.

One condition in which PEM plays an important role is ME/CFS. However, the updated 2021 NICE guideline for ME/CFS no longer recommends graded exercise therapy (GET) [61] anymore (compared to the previous guideline version). GET is defined as “first establishing an individual's baseline of achievable exercise or physical activity, then making fixed incremental increases in the time spent being physically active including supervision by a physiotherapist in a ME/CFS specialist team” [6]. Up to now, studies that investigated GET as an intervention in patients with ME/CFS used also very heterogeneous training approaches (i.e. intensity at 50% of peak heart rate, at 70% from the anaerobic threshold, or just the advice to “start at a level that patients think they can do”) [62]. This paradigm change in the NICE guideline regarding GET evolved also into a controversial debate on the role of exercise training [63–65]. However, it is regarded as common sense that in patients with PEM, activity management strategies must be carefully customized to reflect the individual needs and limits of each individual [66, 67]. We therefore propose an individualized and symptom-titrated approach rather than GET with a fixed progression of the exercise load in patients with mild/moderate PEM. Our recommendation for pacing (energy envelope maintenance) in severe PEM is based on a recent UK study showing that a structured pacing protocol significantly reduced the incidence of PEM and improved the general condition of patients with long COVID [68]. Screening and scoring PEM was suggested to be a useful procedure for assessing the tolerance of certain interventions in patients with chronic fatigue [69]. Pacing was shown to be associated with better outcomes in the management of people with long COVID [70] and may be especially beneficial for individuals with higher available energy who are pushing themselves beyond their energy limitations [57]. Since people with mild to moderate PEM are at risk of developing more frequent, severe, and long-lasting PEM triggered by daily activities, they need to be carefully guided to remain as active as possible while avoiding “crashes” resulting from too much exertion [57].

One limitation of our literature review is that we used only a single database (PubMed). However, our literature review was not designed to provide evidence of effectiveness. Rather, we wanted to compare approaches to exercise training in people with long COVID, and these were found to be very heterogeneous. Another limitation of our proposal is that evidence of the efficacy of our recommendations in people with long COVID is limited so far. Moreover, different recommendations might be necessary for children with long COVID. However, several individual components of our practical recommendations have already been investigated in specific clinical trials and were found to be beneficial and safe in many people with long COVID (e.g. interval endurance training [38] or inspiratory muscle training [30]).

## Conclusion

In our best-practice proposal, we merged the scientific literature and international expert experiences to propose a more homogeneous exercise training concept in people with long COVID, stratified depending on the presence and severity of PEM. These recommendations may guide allied healthcare professionals worldwide to initiate and adjust exercise training programs in long COVID.

### Supplementary Information

**Additional file 1.** Supplementary Material Appendix S1.

## Data Availability

All data generated during the current study (systematic review and online survey) are available in the manuscript and/or the online supplement.

## Abbreviations

6MWT: 6-Minute walk test
CT: Continuous endurance training
DSQ-PEM: DePaul Symptom Questionnaire-Post-Exertional Malaise
HR: Heart rate
HRR: Heart rate reserve
IMT: Inspiratory muscle training
IT: Interval endurance training
ME/CFS: Myalgic encephalomyelitis/chronic fatigue syndrome
NA: Not applicable
PImax: Maximal inspiratory pressure
PEM: Post-exertional malaise
PWR: Peak work rate
Reps: Repetitions
Ref: References
RM: Repetition maximum
Sec: Seconds
WHO: World Health Organization
