# Supplementary material for: Practical Recommendations for Exercise Training in Patients with Long COVID with or without Post-exertional Malaise: A Best Practice Proposal
Source: Sports Med Open. 2024 Apr 24;10:47. doi: 10.1186/s40798-024-00695-8 (PMC11043268; doi:10.1186/s40798-024-00695-8)

**Sports Medicine Open**

**SUPPLEMENTARY MATERIAL**

**Title: Practical recommendations for exercise training in patients with long COVID with or without post-exertional malaise – A best practice proposal**

**Authors:** Rainer Gloeckl^1,2^, Ralf H. Zwick³, Ulrich Fürlinger³, Tessa Schneeberger^1,2^, Daniela Leitl^1,2^, Inga Jarosch^1,2^, Uta Behrends^4,5,6^, Carmen Scheibenbogen^7^, Andreas Rembert Koczulla^1,2,8^

**Affiliations:**

^1^ Institute for Pulmonary Rehabilitation Research, Schoen Klinik Berchtesgadener Land, Schoenau am Koenigssee, Germany

^2^ Department of Pulmonary Rehabilitation, Philipps-University of Marburg, Marburg, Germany

^3^ Therme Wien Med, Ludwig Boltzmann Institute for Rehabilitation Research, Vienna, Austria

^4^ Childrens' Hospital, School of Medicine, Technical University of Munich, Munich, Germany

^5^ German Center for Infection Research (DZIF), Berlin, Germany.

^6^ AGV Research Unit Gene Vectors, Helmholtz Munich (HMGU), Munich, Germany

^7^ Institute of Medical Immunology, Charité - Universitätsmedizin Berlin, corporate member of Freie Universität Berlin and Humboldt Universität zu Berlin, Berlin, Germany

^8^ Teaching Hospital, Paracelsus Medical University Salzburg, Salzburg, Austria

**Corresponding author**

Rainer Gloeckl PhD; Institute for Pulmonary Rehabilitation Research, Schoen Klinik Berchtesgadener Land, Malterhöh 1, 83471 Schoenau am Koenigssee, Germany; Email: rgloeckl@schoen-klinik.de

ORCID: 0000-0002-2741-2748

**
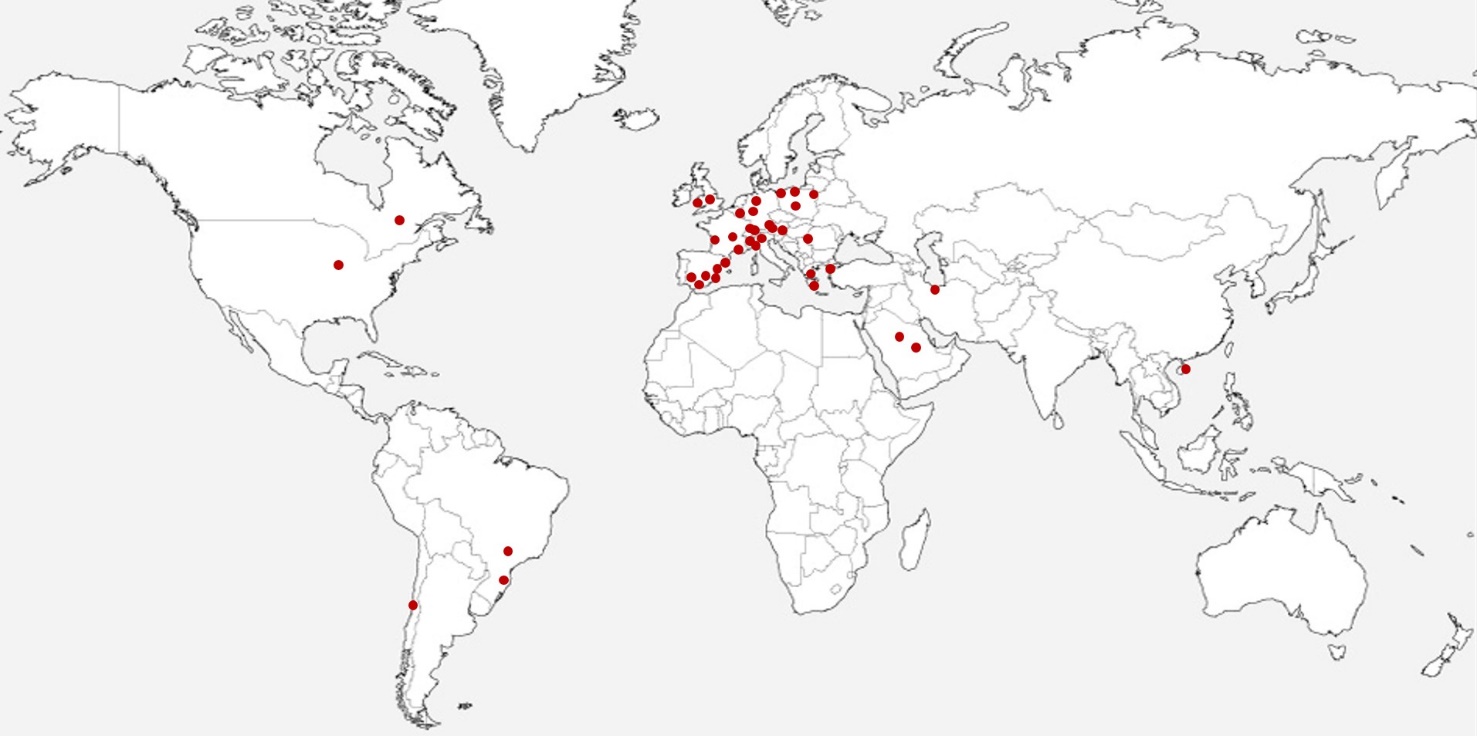
**

**World map on origins of included studies from the literature search**

**Table S1. Additional information on included studies from the literature review**

| **Author** | **Reference** | **Country** | | **City** | | | **Duration of exercise program**  **(sessions**  **per week)** | **Duration of post-COVID symptoms before study inclusion** | **Content of**  **endurance**  **training** | **Content of**  **strength training** |
| --- | --- | --- | --- | --- | --- | --- | --- | --- | --- | --- |
| Liu et al. 2020 | [[1](#_ENREF_1)] | China | | Haikou | | | 6 weeks  (2x/week) | Not reported | N.A. | N.A. |
| Gloeckl et al. 2021 | [[2](#_ENREF_2)] | Germany | | Schönau am Königssee | | | 3 weeks (5x/week) | >6 weeks | Bicycle | Resistance training machines |
| Daynes et al. 2021 | [[3](#_ENREF_3)] | United Kingdom | | Leicester | | | 6 weeks (2x/week) | Not reported | Treadmill | Upper and lower limbs |
| Abodonya et al. 2021 | [[4](#_ENREF_4)] | Saudi Arabia | | Al-Kharj | | | 2 weeks (5x/week) | Not reported | N.A. | N.A. |
| Dalbosco-Salas et al. 2021 | [[5](#_ENREF_5)] | Chile | | Santiago | | | 9 weeks (2-3x/week) | 30 (27-35) days | Not reported | Not reported |
| Martin et al. 2021 | [[6](#_ENREF_6)] | Belgium | | Brussels | | | 6 weeks (2x/week) | Not reported | Not reported | Upper and lower body muscles with material available at home (e.g. water bottle, chair) |
| Nambi et al. 2021 | [[7](#_ENREF_7)] | Saudi Arabia | | Al-Kharj | | | 8 weeks (4x/week) | Not reported | Treadmill, bicycle | Weights |
| Stavrou et al. 2021 | [[8](#_ENREF_8)] | Greece | | Larissa | | | 8 weeks (3x/week) | 2 months | Walking | Dumbbell side lateral raises and squats, chair lunges, Seated leg raises, Elbow flexion-extension on the chest with medicine ball |
| Mohamed et al. 2021 | [[9](#_ENREF_9)] | Turkey | | Istanbul | | | 2 weeks (3x/week) | Not reported | Walking, treadmill, bicycle | N.A. |
| Betschart et al. 2021 | [[10](#_ENREF_10)] | Switzerland | | Winterthur | | | 2xweek | Median 73 days | Bicycle | Not reported |
| Hayden et al. 2021 | [[11](#_ENREF_11)] | Germany | | Bad Reichenhall | | | 3 weeks (endurance 3-5x/week, strength 2-3x/week) | >4 weeks | Bicycle, Nordic Walking | Strength training machines |
| Spielmanns et al. 2021 | [[12](#_ENREF_12)] | Switzerland | | Wald | | | 3 weeks  (5-6x/week) | Not reported | Bicycle, treadmill | Not reported |
| Udina et al. 2021 | [[13](#_ENREF_13)] | Spain | | Barcelona | | | Variable, median 8,2 +/- 1,7 days) (7x/week) | Not reported | Bicycle, steps or walking | Upper and lower limb functional exercise |
| **Author** | **Reference** | **Country** | | **City** | | | **Duration of exercise program**  **(sessions**  **per week)** | **Duration of post-COVID symptoms before study inclusion** | **Content of**  **endurance**  **training** | **Content of**  **strength training** |
| Zampogna et al. 2021 | [[14](#_ENREF_14)] | Italy | | Tradate | | | Not reported | Not reported | Bicycle | Without devices or light weights bands, canes, balls |
| Bouteleux et al. 2021 | [[15](#_ENREF_15)] | France | | Talence | | | Variable, median 66 days (3x/week) | Median 73 days | Not reported | Not reported |
| Albu et al. 2021 | [[16](#_ENREF_16)] | Spain | | Badalona | | | 8weeks or longer (5x/week) | >12 weeks | Bicycle | Submaximal static and dynamic exercise |
| Al Chikhanie et al. 2021 | [[17](#_ENREF_17)] | France | | Dieulefit | | | 27,6 +/- 14,2 days | Not reported | Not reported | Not reported |
| Besnier et al. 2022 | [[18](#_ENREF_18)] | Canada | | Montréal | | | 8 weeks (3x/week) | >12 weeks | Bicycle | Weight machines, free weights, elastic bands |
| Jimeno-Almazan et al. 2022 | [[19](#_ENREF_19)] | Spain | | Murcia | | | 8 weeks (3x/week) | >12 weeks | Not reported | Squat, bench press, deadlift and bench pull |
| Li et al. 2022 | [[20](#_ENREF_20)] | China | | Sichuan | | | 6 weeks  (3-4x/week) | >6 weeks | Not reported | Not reported |
| Capin et al. 2022 | [[21](#_ENREF_21)] | United States | | Aurora | | | 12 weeks (1x/week) | <6 weeks | Walking, cycling, rowing; includes low-intensity endurance and high-intensity interval training | Body-weight exercises |
| McNarry et al. 2022 | [[22](#_ENREF_22)] | United Kingdom | | Swansea | | | 8 weeks (3x/week) | Median 9+/- 4,2 months | N.A. | N.A. |
| Nopp et al. 2022 | [[23](#_ENREF_23)] | Austria | | Vienna | | | 6 weeks (3x/week) | Median 4,4+/-2 months | Not reported | Not reported |
| Contreras-Briceno et al. 2022 | [[24](#_ENREF_24)] | Chile | | Santiago | | | Not reported | Not reported | N.A. | N.A. |
| Hockele et al. 2022 | [[25](#_ENREF_25)] | Brazil | | Caxias do Sul | | | 8 weeks (2xweek) | Not reported | Treadmill, Bicycle | Dumbbells (2-5kg), ankle weight (2-5kg), theraband |
| Teixeira do Amaral et al. 2022 | [[26](#_ENREF_26)] | Brazil | | Bauru | | | 12 weeks (3-5x/week) | 30-45days | Walking, Bicycle | Body weight exercises for upper and lower extremities |
|  | |  |  | |  |  | | | | |
| **Author** | **Reference** | **Country** | | **City** | | | **Duration of exercise program**  **(sessions**  **per week)** | **Duration of post-COVID symptoms before study inclusion** | **Content of**  **endurance**  **training** | **Content of**  **strength training** |
| Palau et al. 2022 | [[27](#_ENREF_27)] | Spain | | Valencia | | | 12 week (2x/day) | >12 weeks | N.A. | N.A. |
| Estebanez-Pérez et al. 2022 | [[28](#_ENREF_28)] | Spain | | Málaga | | | 4 weeks (3-5x/week) | >8weeks | Individualised | Individualised (e.g. glute bridge, spine curl) |
| Rutkowski et al. 2022 | [[29](#_ENREF_29)] | Poland | | Opole | | | 3 weeks (5x/week) | Not reported | Bicycle egometer | Not reported |
| Corna et al. 2022 | [[30](#_ENREF_30)] | Italy | | Gattico-Veruno | | | 2 weeks (5x/week) | <100 days | Walking, arm crank ergometer | Upper and lower limb  strengthening |
| Vitacca et al. 2022 | [[31](#_ENREF_31)] | Italy | | Lumezzane | | | Not reported | 4-12 weeks | Bicycle | N.A. |
| Asimakos et al. 2023 | [[32](#_ENREF_32)] | Greece | | Athens | | | 8 weeks (2x/week) | 6-8 weeks post hospital discharge | Bicycle | Knee extension, seated chest press, peck deck, lateral pull-down |
| Ostrowska et al. 2023 | [[33](#_ENREF_33)] | Poland | | Bydgoszcz | | | 6 weeks (3x/week) | 2-12 months | Not reported | Not reported |
| Jimeno-Almazan et al. 2023 | [[34](#_ENREF_34)] | Spain | | Murcia | | | 8 week (3x/week) | >12 weeks | Not reported | Squat, bench press, deadlift, and bench pull |
| Spielmanns et al. 2023 | [[35](#_ENREF_35)] | Switzerland | | Wald | | | 3 weeks | Not reported | Bicycle, treadmill, walking | Not reported |
| Colas et al. 2023 | [[36](#_ENREF_36)] | France | | Saint-Etienne | | | 4 weeks (2hx/week) | >12 weeks | Not reported | Not reported |
| Alsharidah et al. 2023 | [[37](#_ENREF_37)] | Saudi Arabia | | Buraidah | | | 6 weeks (3x/week) | Not  reported | Walking, running (outside or treadmill) | Weights |
| Ghasemi et al. 2023 | [[38](#_ENREF_38)] | Iran | | Tehran | | | 4 weeks  (3x/week) | Not  reported | N.A. | Resistance Band and body-weight (Plank, Jumping Jack, Sit-ups) |
| Minko et al. 2023 | [[39](#_ENREF_39)] | Poland | | Szczecin | | | 2-6 weeks (6x/week) | <12 months | Stair climbing, outdoor walking, bicycle | Not reported |
| Espinoza-Bravo et al. 2023 | [[40](#_ENREF_40)] | Chile | | Santiago | | | 8 weeks  (3x/week) | >6 weeks | Walking | Body Weight or low weights |
| **Author** | **Reference** | **Country** | | **City** | | | **Duration of exercise program**  **(sessions**  **per week)** | **Duration of post-COVID symptoms before study inclusion** | **Content of**  **endurance**  **training** | **Content of**  **strength training** |
| Mooren et al. 2023 | [[41](#_ENREF_41)] | Germany | | Witten | | | 4-6 weeks  (3-5x/ week) | >12 weeks | Bicycle, aqua fitness, walking, circuit training, aerobic group exercise | Not reported |
| Del Corral et al. 2023 | [[42](#_ENREF_42)] | Spain | | Madrid | | | 8 weeks  (6x/week) | >12 weeks | N.A. | N.A. |
| Rodriguez-Blanco et al. 2023 | [[43](#_ENREF_43)] | Spain | | Sevilla | | | 2 weeks  (7x/week) | >4 weeks | N.A. | Not reported |
| Romanet et al. 2023 | [[44](#_ENREF_44)] | France | | Paris | | | 10 weeks  (2x/week) | >12 weeks | Bicycle | Steppers, rowing machines, treadmills, weights (upper and lower limb) |
| Kerling et al. 2024 | [[45](#_ENREF_45)] | Germany | | Hannover | | | 12 weeks (3x/week) | Not  reported | Individual plan  (e.g. cycling, walking, indoor cycling, cross-training, swimming, jogging) | Individual plan (e.g. equipment-based training, single-limb strength training, stability  training or fitness videos) |
| Pietranis et al. 2024 | [[46](#_ENREF_46)] | Poland | | Bialystok | | | 6 weeks  (3x/week) | Not  reported | Bicycle | Major muscle groups (e.g., weight training on machines, free weights, elastic resistance bands) |

*Abbreviations: CT – continuous endurance training, HR – heart rate, HRR – heart rate reserve, IMT – inspiratory muscle training, IT – interval endurance training, MIP – maximum inspiratory pressure, NA – not applied, PImax – maximal inspiratory pressure, PEM – post-exertional malaise, PWR – peak work rate, Ref – reference, RM – repetition maximum, reps – repetitions, sec – seconds*

**Online Supplement References**

1. Liu, K., et al., *Respiratory rehabilitation in elderly patients with COVID-19: A randomized controlled study.* Complementary therapies in clinical practice, 2020. **39**: p. 101166.

2. Gloeckl, R., et al., *Benefits of pulmonary rehabilitation in COVID-19: a prospective observational cohort study.* ERJ open research, 2021. **7**(2): p. 00108-2021.

3. Daynes, E., et al., *Early experiences of rehabilitation for individuals post-COVID to improve fatigue, breathlessness exercise capacity and cognition - A cohort study.* Chronic respiratory disease, 2021. **18**: p. 14799731211015691.

4. Abodonya, A.M., et al., *Inspiratory muscle training for recovered COVID-19 patients after weaning from mechanical ventilation: A pilot control clinical study.* Medicine, 2021. **100**(13): p. e25339.

5. Dalbosco-Salas, M., et al., *Effectiveness of a Primary Care Telerehabilitation Program for Post-COVID-19 Patients: A Feasibility Study.* Journal of clinical medicine, 2021. **10**(19).

6. Martin, I., et al., *Follow-up of functional exercise capacity in patients with COVID-19: It is improved by telerehabilitation.* Respiratory medicine, 2021. **183**: p. 106438.

7. Nambi, G., et al., *Comparative effectiveness study of low versus high-intensity aerobic training with resistance training in community-dwelling older men with post-COVID 19 sarcopenia: A randomized controlled trial.* Clinical rehabilitation, 2022. **36**(1): p. 59-68.

8. Stavrou, V.T., et al., *Eight Weeks Unsupervised Pulmonary Rehabilitation in Previously Hospitalized of SARS-CoV-2 Infection.* Journal of personalized medicine, 2021. **11**(8).

9. Mohamed, A.A. and M. Alawna, *The effect of aerobic exercise on immune biomarkers and symptoms severity and progression in patients with COVID-19: A randomized control trial.* Journal of bodywork and movement therapies, 2021. **28**: p. 425-432.

10. Betschart, M., et al., *Feasibility of an Outpatient Training Program after COVID-19.* International journal of environmental research and public health, 2021. **18**(8).

11. Hayden, M.C., et al., *Effectiveness of a Three-Week Inpatient Pulmonary Rehabilitation Program for Patients after COVID-19: A Prospective Observational Study.* International journal of environmental research and public health, 2021. **18**(17).

12. Spielmanns, M., et al., *Effects of a Comprehensive Pulmonary Rehabilitation in Severe Post-COVID-19 Patients.* Int. J. Environ. Res. Public Health, 2021. **18**(2695).

13. Udina, C., et al., *Rehabilitation in adult post-COVID-19 patients in post-acute care with Therapeutic Exercise.* The Journal of frailty & aging, 2021. **10**(3): p. 297-300.

14. Zampogna, E., et al., *Pulmonary Rehabilitation in Patients Recovering from COVID-19.* Respiration; international review of thoracic diseases, 2021. **100**(5): p. 416-422.

15. Bouteleux, B., et al., *Respiratory rehabilitation for Covid-19 related persistent dyspnoea: A one-year experience.* Respiratory medicine, 2021. **189**: p. 106648.

16. Albu, S., et al., *Multidisciplinary outpatient rehabilitation of physical and neurological sequelae and persistent symptoms of covid-19: a prospective, observational cohort study.* Disability and rehabilitation, 2022. **44**(22): p. 6833-6840.

17. Al Chikhanie, Y., et al., *Effectiveness of pulmonary rehabilitation in COVID-19 respiratory failure patients post-ICU.* Respiratory physiology & neurobiology, 2021. **287**: p. 103639.

18. Besnier, F., et al., *Cardiopulmonary Rehabilitation in Long-COVID-19 Patients with Persistent Breathlessness and Fatigue: The COVID-Rehab Study.* International journal of environmental research and public health, 2022. **19**(7).

19. Jimeno-Almazán, A., et al., *Rehabilitation for post-COVID-19 condition through a supervised exercise intervention: A randomized controlled trial.* Scand J Med Sci Sports, 2022. **32**: p. 1797-1801.

20. Li, J., et al., *A telerehabilitation programme in post-discharge COVID-19 patients (TERECO): a randomised controlled trial.* Thorax, 2022. **77**(7): p. 697-706.

21. Capin, J.J., et al., *Safety, feasibility and initial efficacy of an app-facilitated telerehabilitation (AFTER) programme for COVID-19 survivors: a pilot randomised study.* BMJ open, 2022. **12**(7): p. e061285.

22. McNarry, M.A., et al., *Inspiratory muscle training enhances recovery post-COVID-19: a randomised controlled trial.* The European respiratory journal, 2022. **60**(4).

23. Nopp, S., et al., *Outpatient Pulmonary Rehabilitation in Patients with Long COVID Improves Exercise Capacity, Functional Status, Dyspnea, Fatigue, and Quality of Life.* Respiration; international review of thoracic diseases, 2022. **101**(6): p. 593-601.

24. Contreras-Briceno, F., et al., *Eccentric Training in Pulmonary Rehabilitation of Post-COVID-19 Patients: An Alternative for Improving the Functional Capacity, Inflammation, and Oxidative Stress.* Biology, 2022. **11**(10).

25. Hockele, L.F., et al., *Pulmonary and Functional Rehabilitation Improves Functional Capacity, Pulmonary Function and Respiratory Muscle Strength in Post COVID-19 Patients: Pilot Clinical Trial.* International journal of environmental research and public health, 2022. **19**(22).

26. Teixeira, D.O.A.V., et al., *Cardiovascular, Respiratory, and Functional Effects of Home-Based Exercise Training after COVID-19 Hospitalization.* Medicine and science in sports and exercise, 2022. **54**(11): p. 1795-1803.

27. Palau, P., et al., *Effect of a home-based inspiratory muscle training programme on functional capacity in postdischarged patients with long COVID: the InsCOVID trial.* BMJ open respiratory research, 2022. **9**(1).

28. Estebanez-Perez, M.J., J.M. Pastora-Bernal, and R. Martin-Valero, *The Effectiveness of a Four-Week Digital Physiotherapy Intervention to Improve Functional Capacity and Adherence to Intervention in Patients with Long COVID-19.* International journal of environmental research and public health, 2022. **19**(15).

29. Rutkowski, S., et al., *Effectiveness of an Inpatient Virtual Reality-Based Pulmonary Rehabilitation Program among COVID-19 Patients on Symptoms of Anxiety, Depression and Quality of Life: Preliminary Results from a Randomized Controlled Trial.* International journal of environmental research and public health, 2022. **19**(24).

30. Corna, S., et al., *Effects of Aerobic Training in Patients with Subacute COVID-19: A Randomized Controlled Feasibility Trial.* International journal of environmental research and public health, 2022. **19**(24).

31. Vitacca, M., et al., *Intermittent versus equivalent constant-load cycle training in COVID-19 patients.* Pulmonology, 2022. **28**(4): p. 312-314.

32. Asimakos, A., et al., *Additive benefit of rehabilitation on physical status, symptoms and mental health after hospitalisation for severe COVID-19 pneumonia.* BMJ open respiratory research, 2023. **10**(1).

33. Ostrowska, M., et al., *Effects of Multidisciplinary Rehabilitation Program in Patients with Long COVID-19: Post-COVID-19 Rehabilitation (PCR SIRIO 8) Study.* Journal of clinical medicine, 2023. **12**(2).

34. Jimeno-Almazan, A., et al., *Effects of a concurrent training, respiratory muscle exercise, and self-management recommendations on recovery from post-COVID-19 conditions: the RECOVE trial.* Journal of applied physiology, 2023. **134**(1): p. 95-104.

35. Spielmanns, M., et al., *Pulmonary Rehabilitation Outcomes of Post-Acute COVID-19 Patients during Different Waves of the Pandemic.* International journal of environmental research and public health, 2023. **20**(10).

36. Colas, C., et al., *Physical Activity in Long COVID: A Comparative Study of Exercise Rehabilitation Benefits in Patients with Long COVID, Coronary Artery Disease and Fibromyalgia.* Int J Environ Res Public Health, 2023. **20**(15).

37. Alsharidah, A.S., et al., *A Pulmonary Telerehabilitation Program Improves Exercise Capacity and Quality of Life in Young Females Post-COVID-19 Patients.* Ann Rehabil Med, 2023. **47**(6): p. 502-510.

38. Ghasemi, M., et al., *Experience with telemedicine in neuromuscular clinic during COVID-19 pandemic.* Acta Myol, 2023. **42**(1): p. 14-23.

39. Minko, A., et al., *Effects of Comprehensive Rehabilitation on Pulmonary Function in Patients Recovering from COVID-19.* Int J Environ Res Public Health, 2023. **20**(5).

40. Espinoza-Bravo, C., et al., *Effectiveness of Functional or Aerobic Exercise Combined With Breathing Techniques in Telerehabilitation for Patients With Long COVID: A Randomized Controlled Trial.* Phys Ther, 2023. **103**(11).

41. Mooren, J.M., et al., *Medical Rehabilitation of Patients with Post-COVID-19 Syndrome-A Comparison of Aerobic Interval and Continuous Training.* J Clin Med, 2023. **12**(21).

42. Del Corral, T., et al., *Home-based respiratory muscle training on quality of life and exercise tolerance in long-term post-COVID-19: Randomized controlled trial.* Ann Phys Rehabil Med, 2023. **66**(1): p. 101709.

43. Rodriguez-Blanco, C., et al., *A 14-Day Therapeutic Exercise Telerehabilitation Protocol of Physiotherapy Is Effective in Non-Hospitalized Post-COVID-19 Conditions: A Randomized Controlled Trial.* J Clin Med, 2023. **12**(3).

44. Romanet, C., et al., *Effectiveness of exercise training on the dyspnoea of individuals with long COVID: A randomised controlled multicentre trial.* Ann Phys Rehabil Med, 2023. **66**(5): p. 101765.

45. Kerling, A., et al., *Effects of a randomized-controlled and online-supported physical activity intervention on exercise capacity, fatigue and health related quality of life in patients with post-COVID-19 syndrome.* BMC Sports Sci Med Rehabil, 2024. **16**(1): p. 33.

46. Pietranis, K.A., et al., *Effects of Pulmonary Rehabilitation on Respiratory Function and Thickness of the Diaphragm in Patients with Post-COVID-19 Syndrome: A Randomized Clinical Trial.* J Clin Med, 2024. **13**(2).

**Original questions and answers from the expert online survey concerning**

**the prescription of exercise training in post-COVID patients**

(n=14 experts responded)
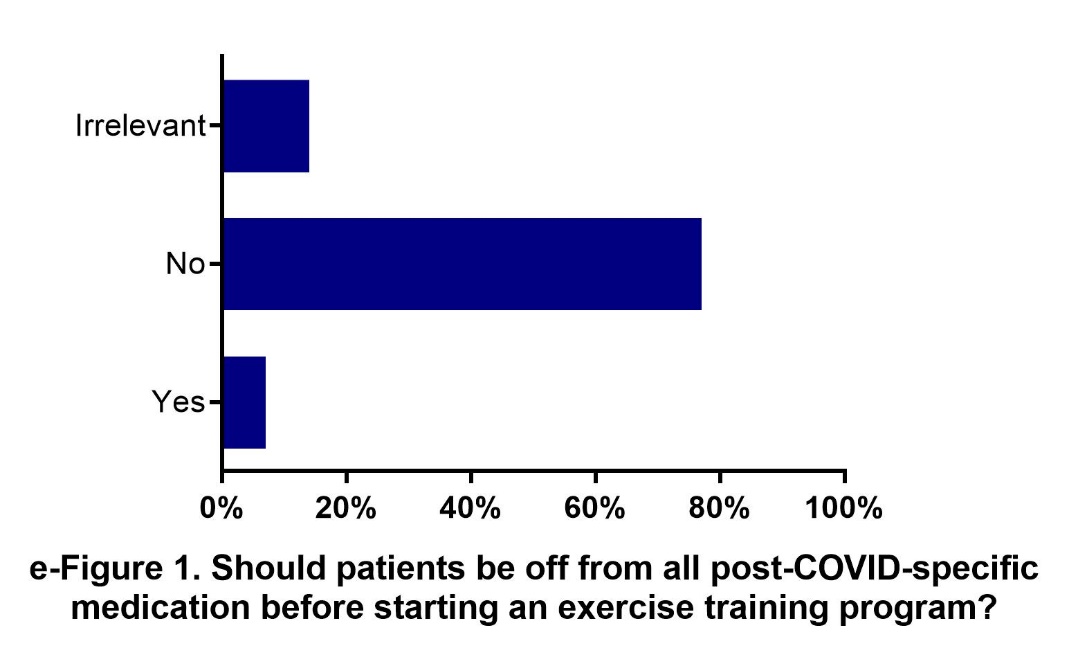

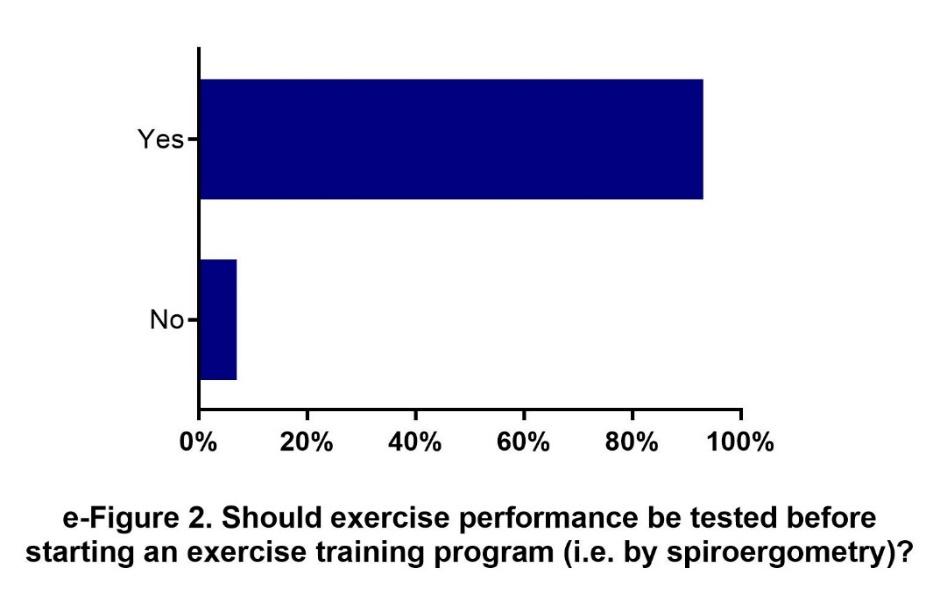

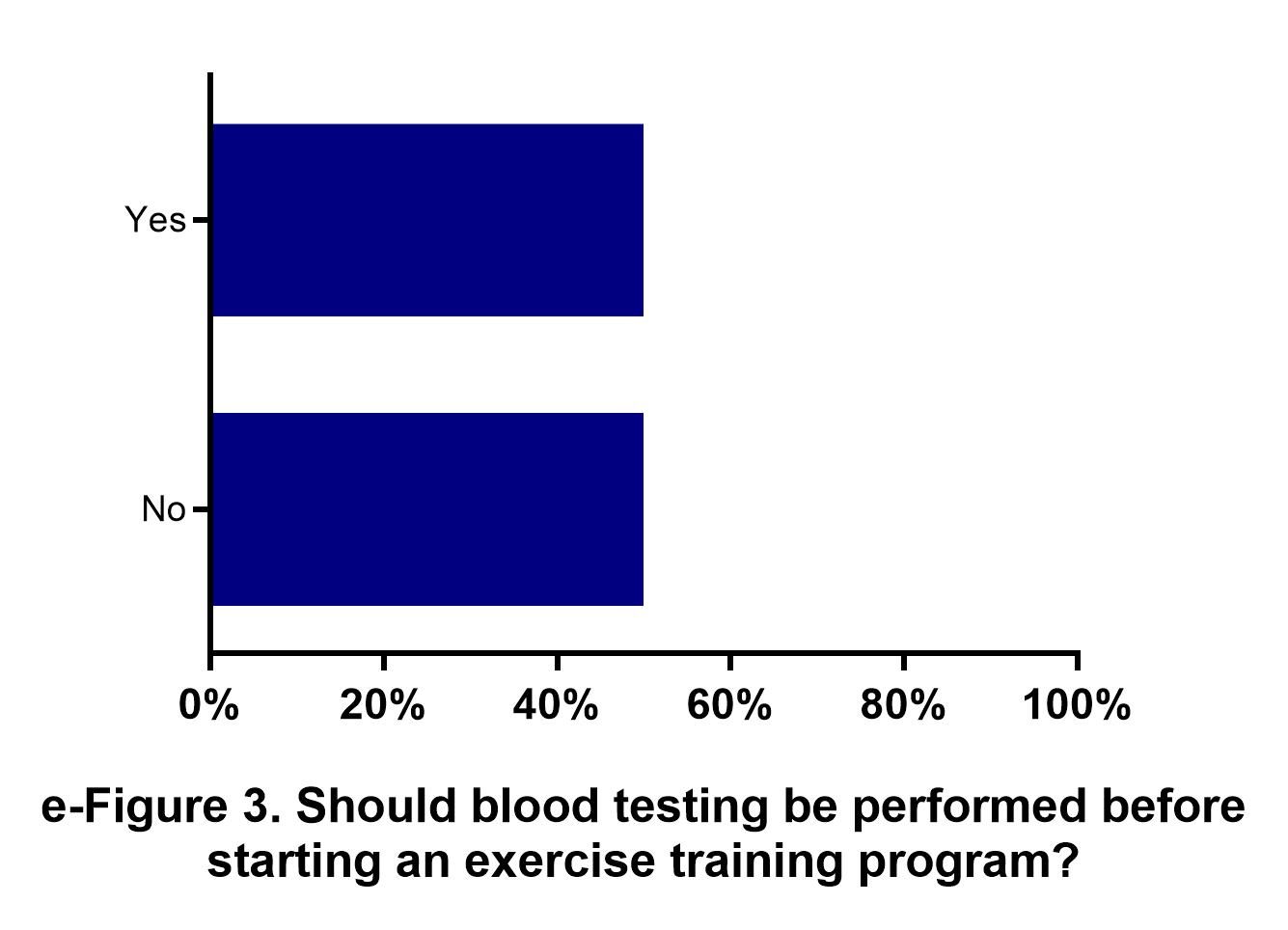

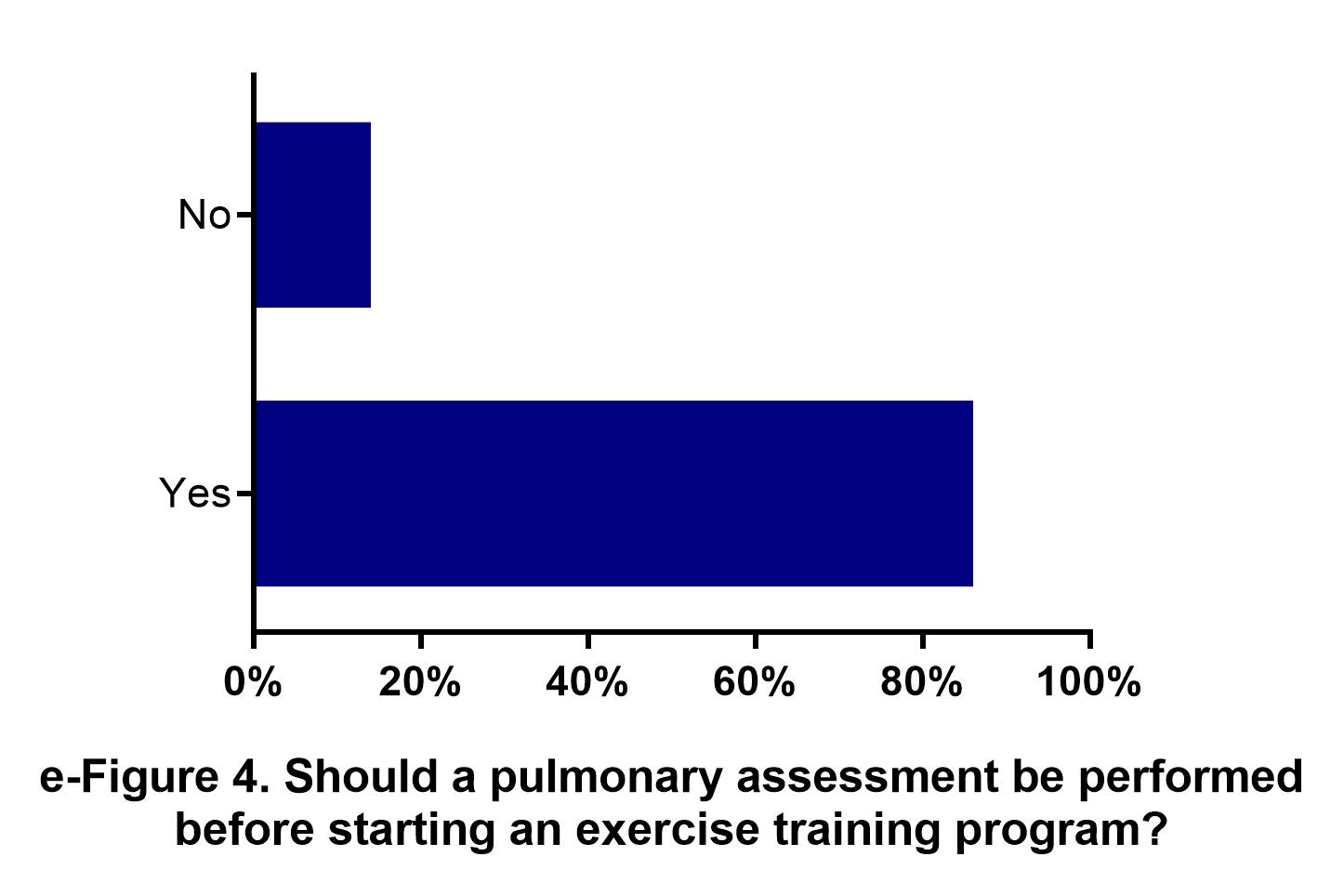

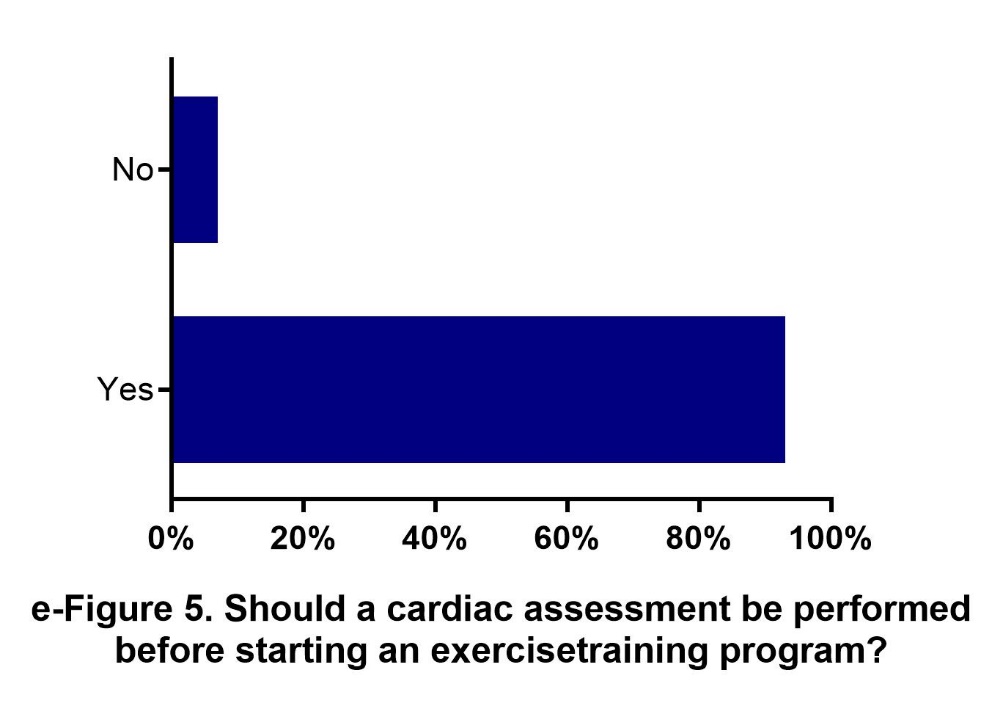

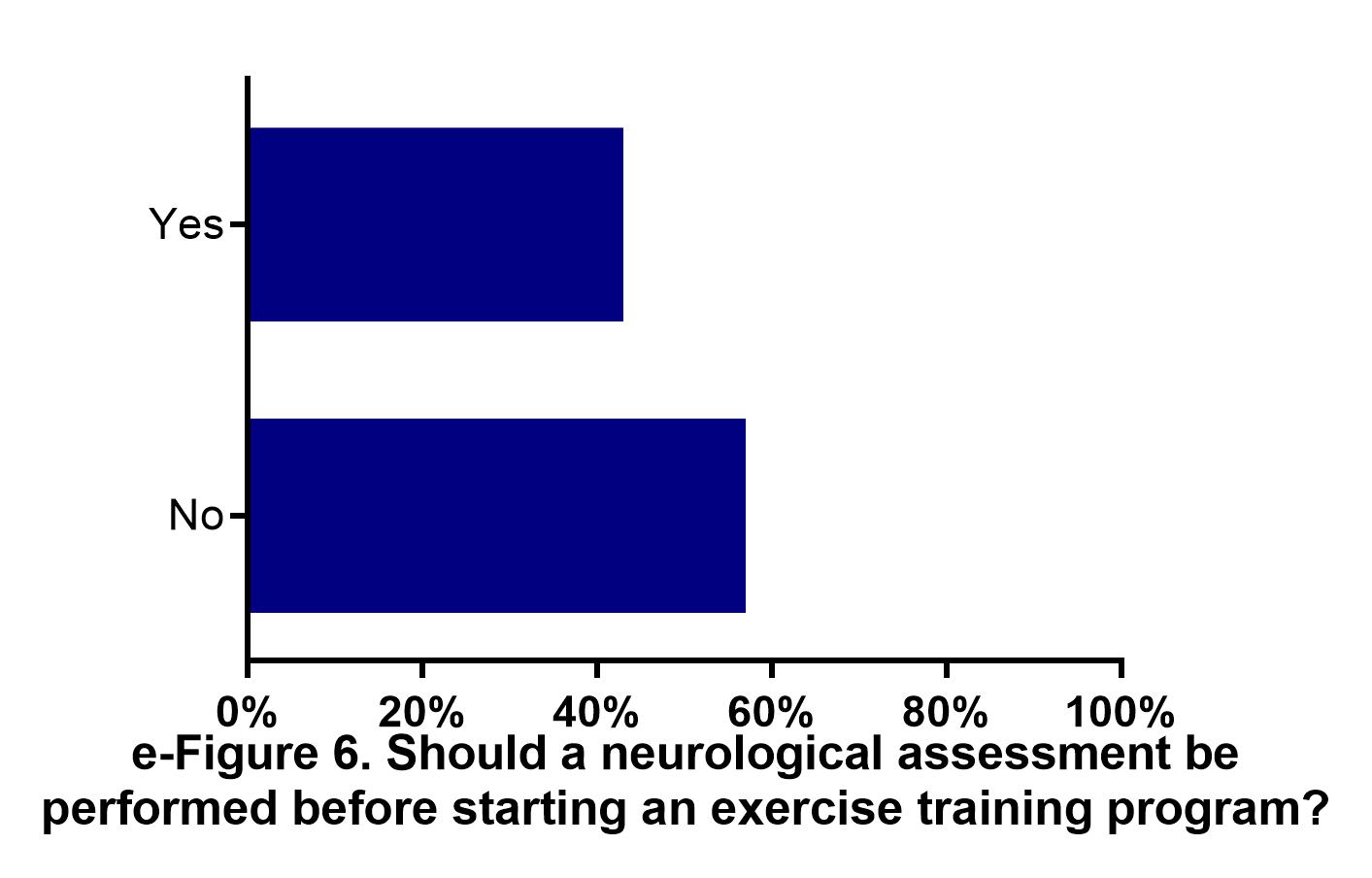

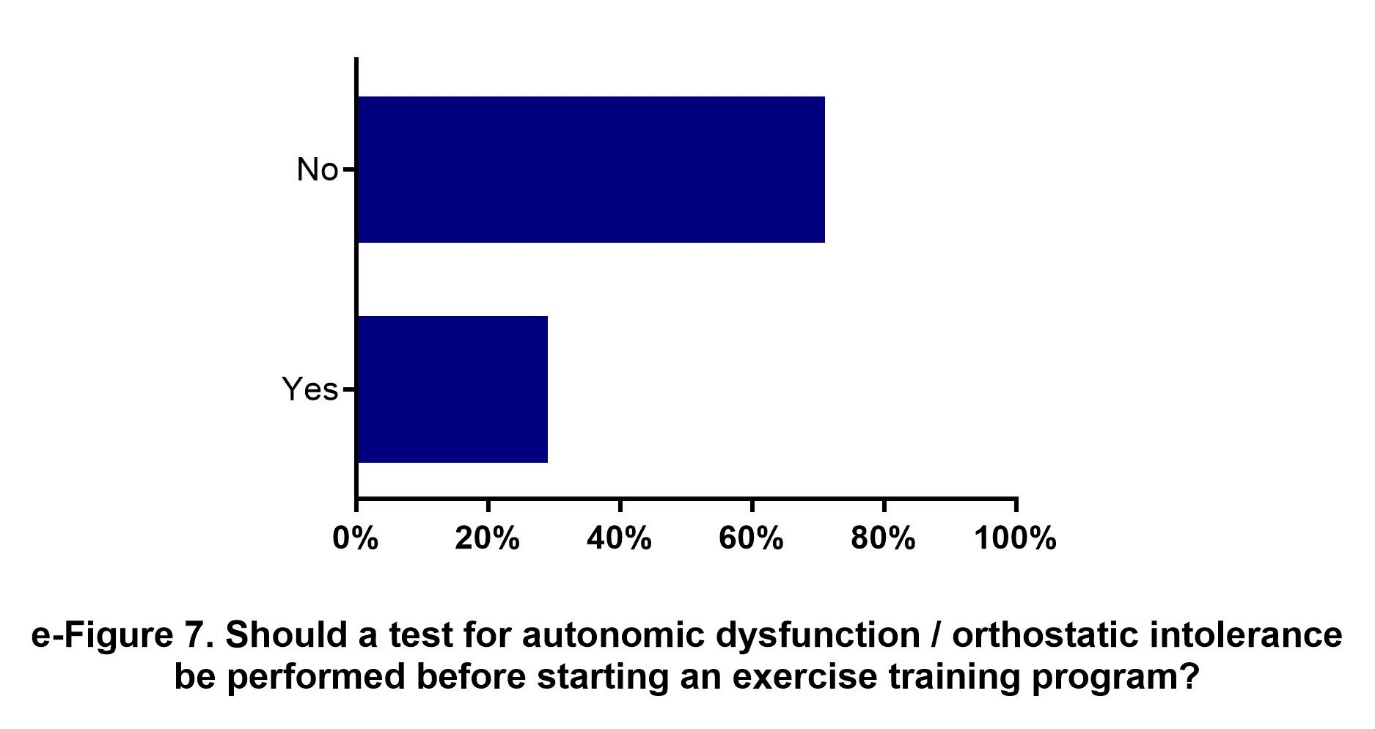

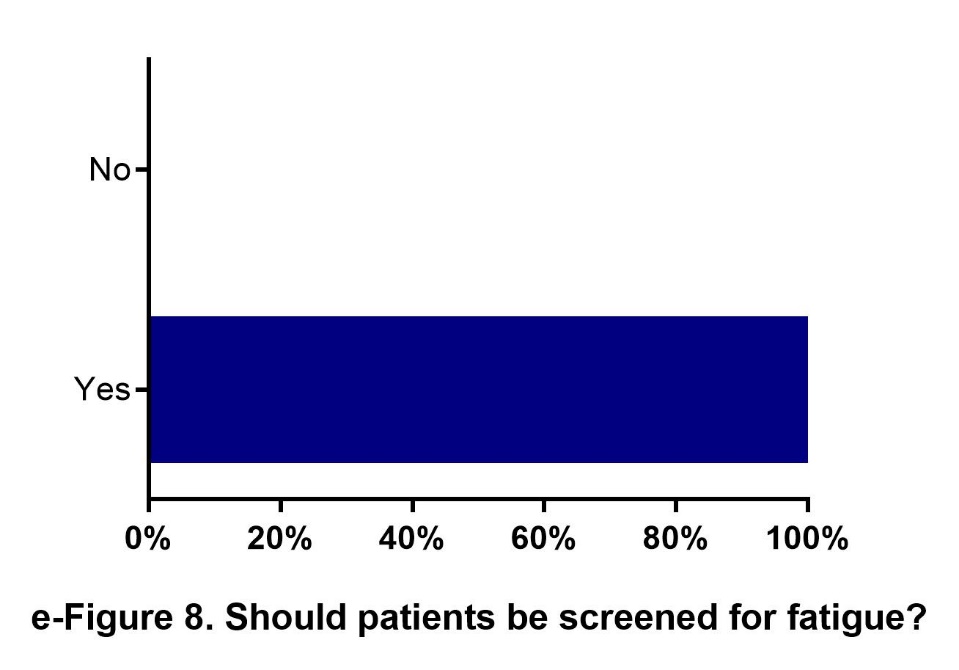

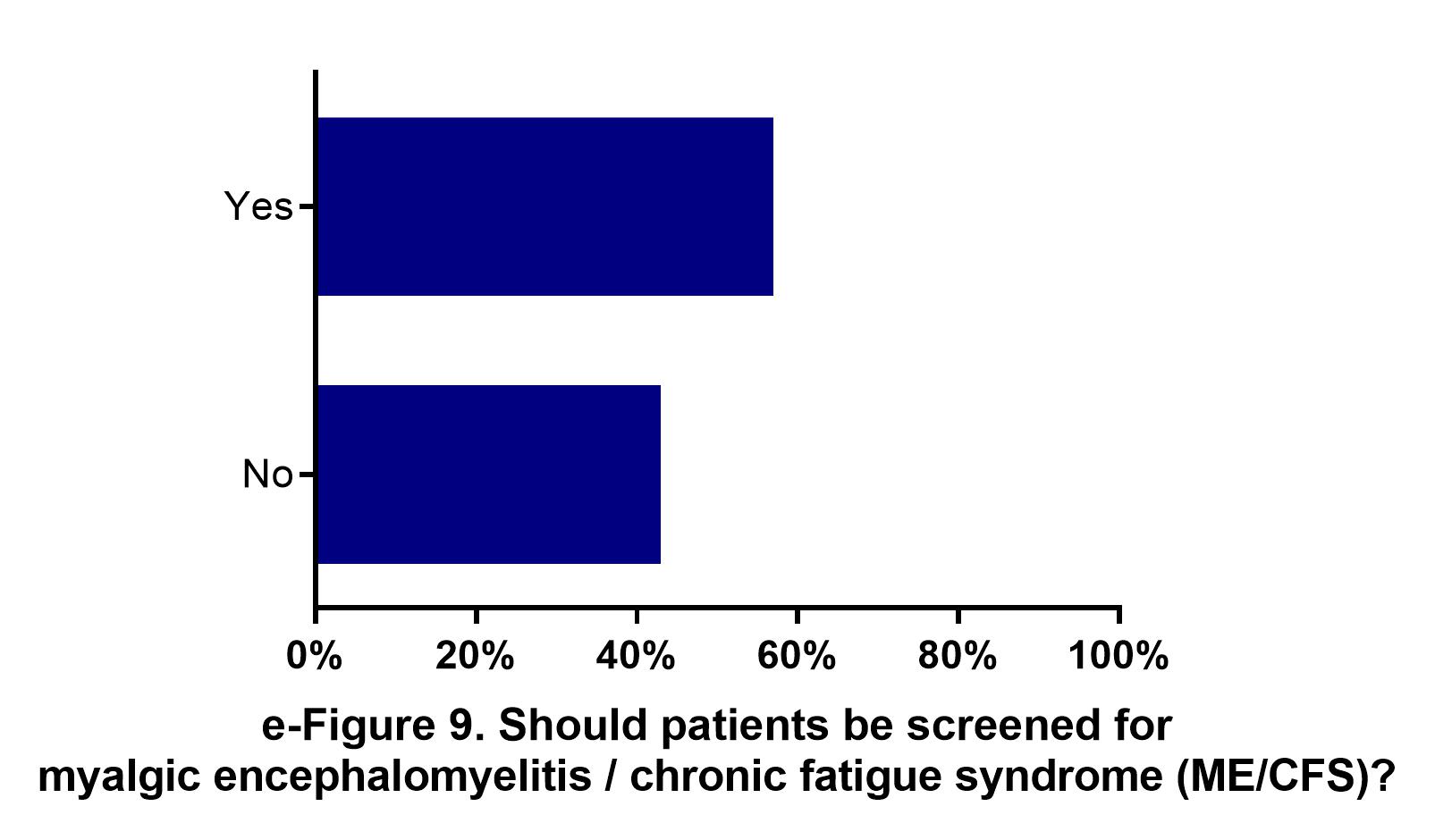

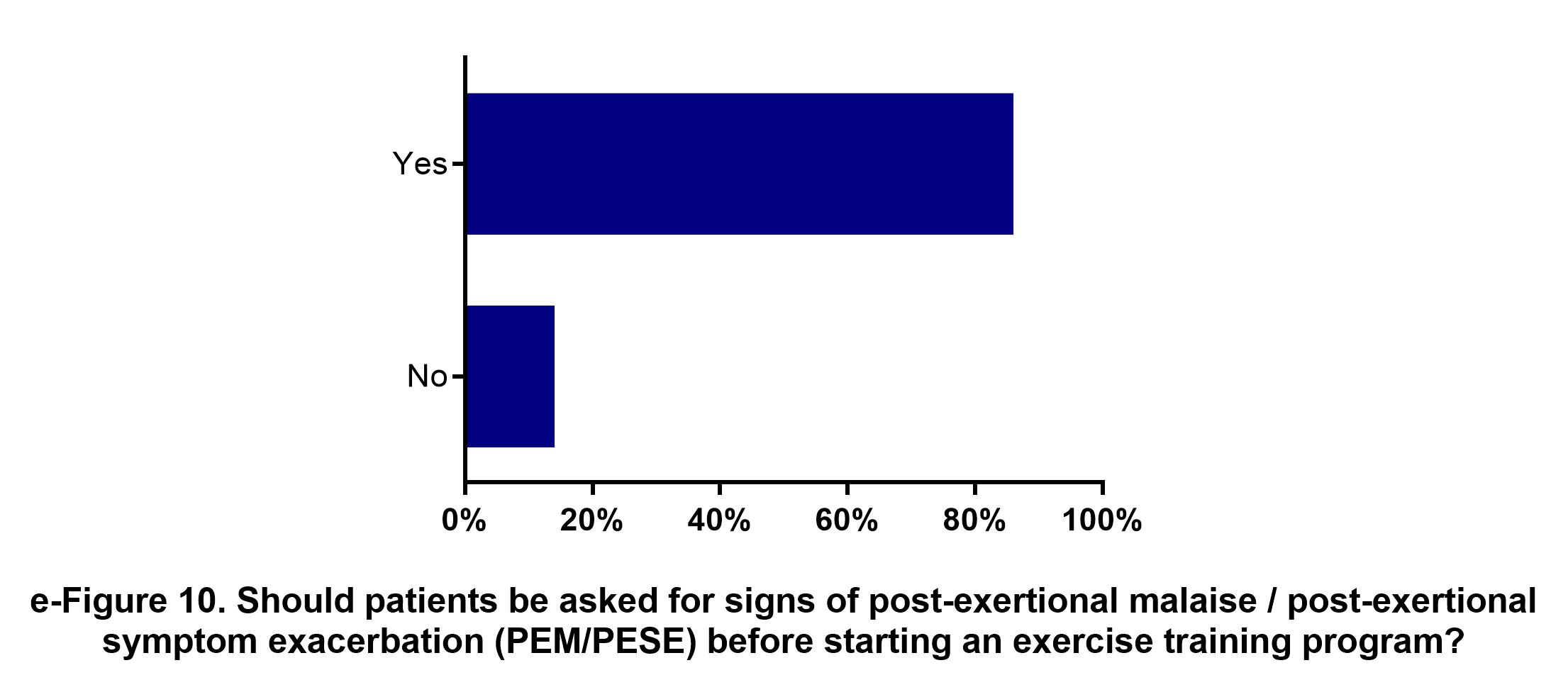

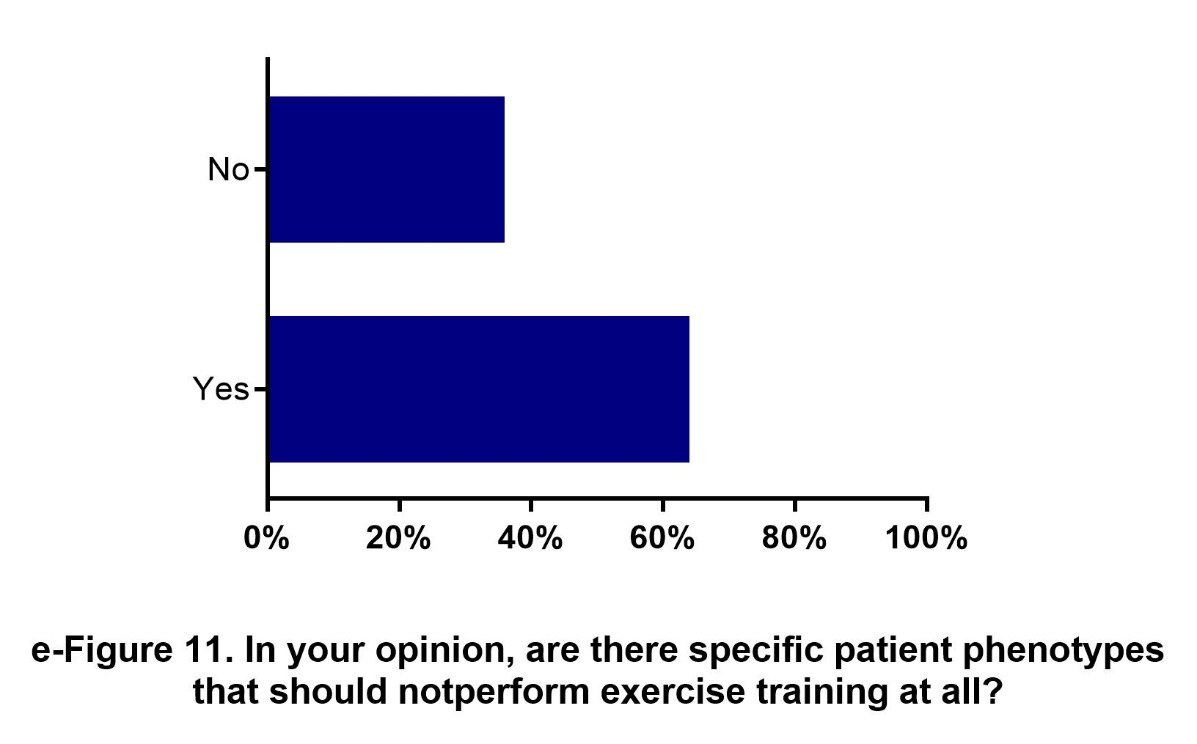

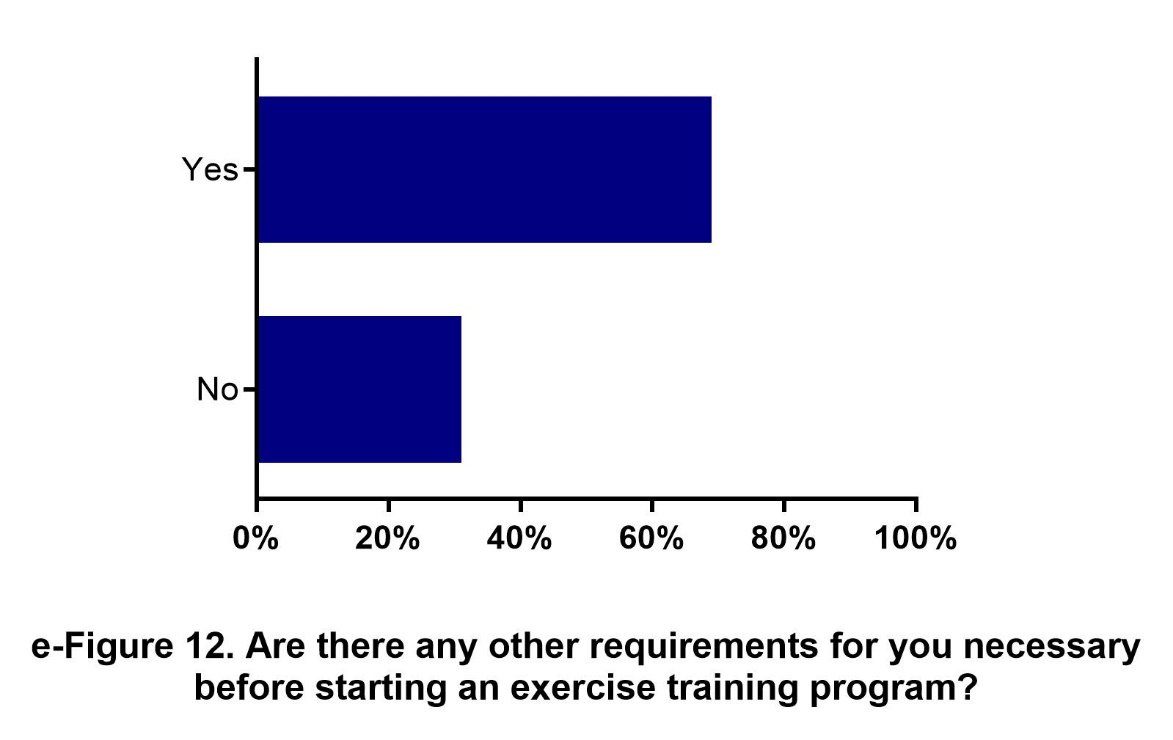

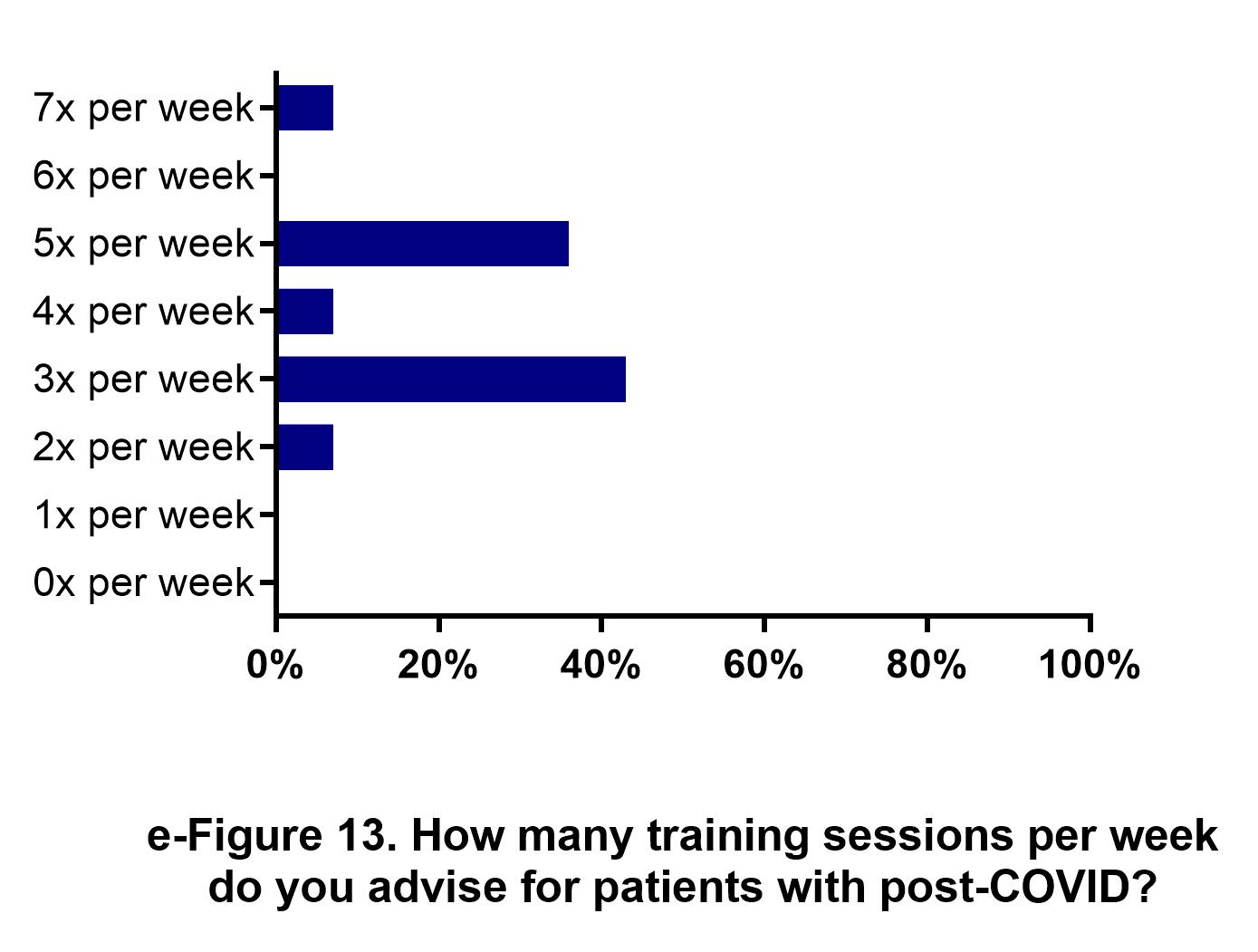

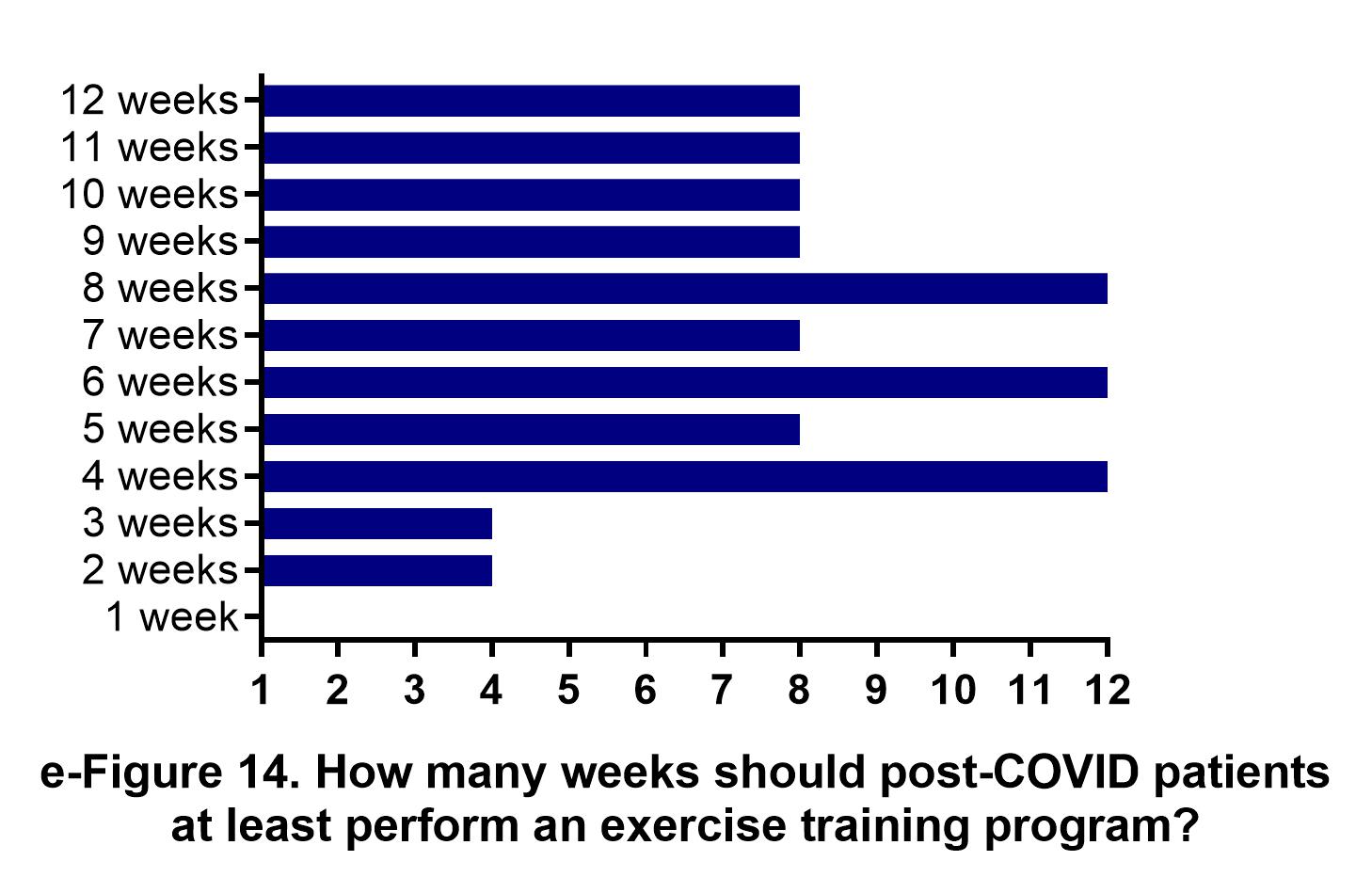

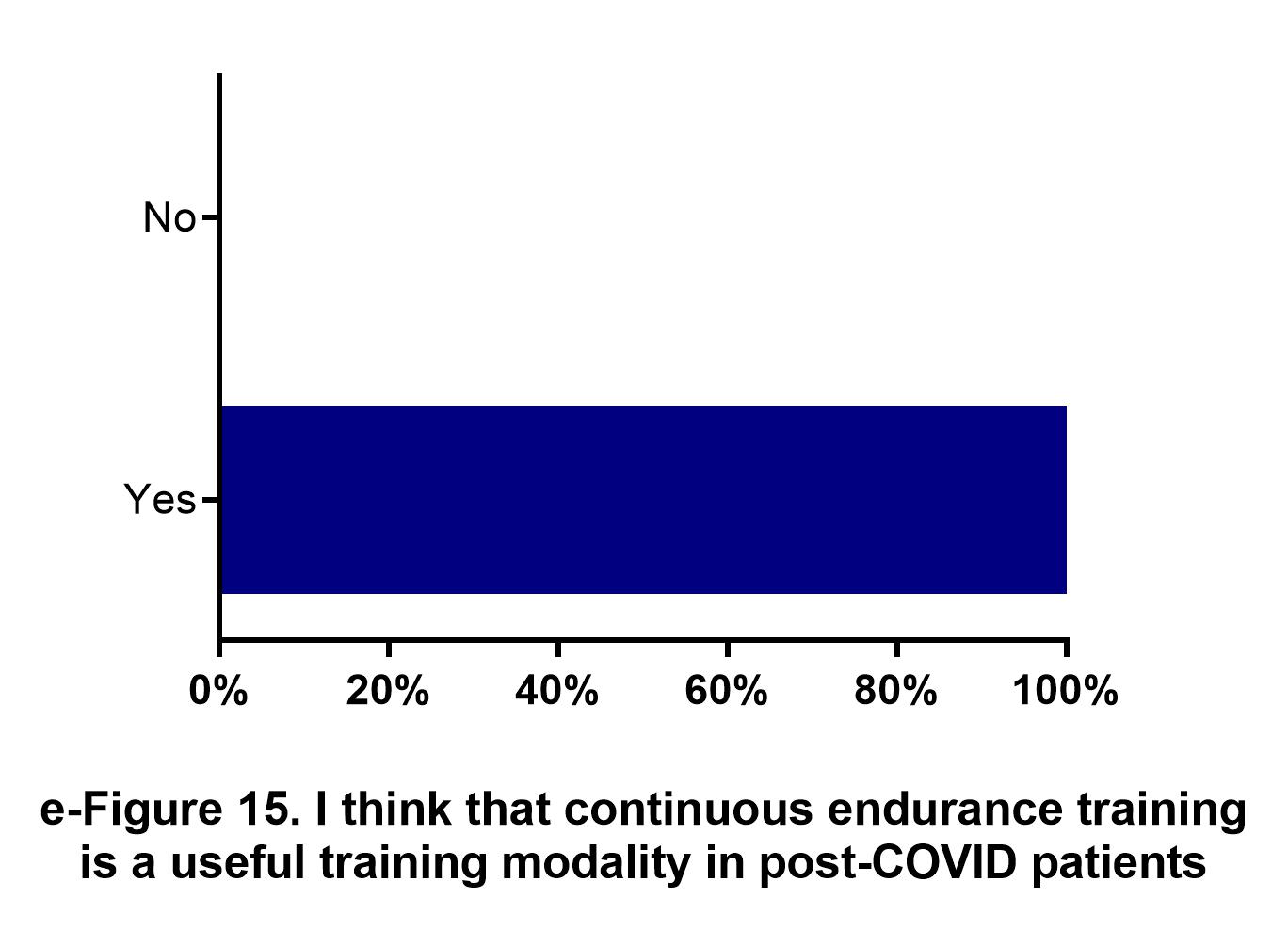

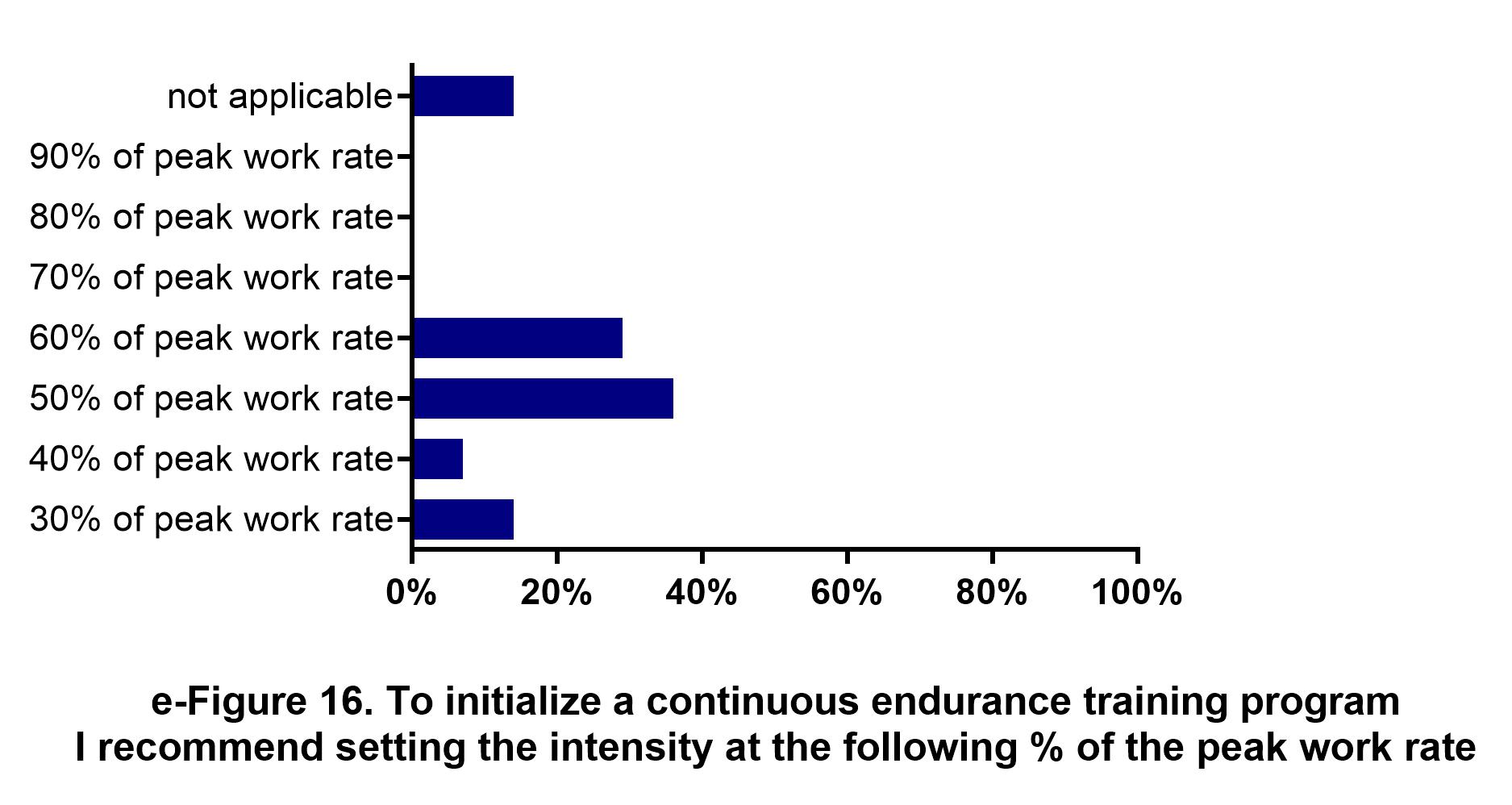

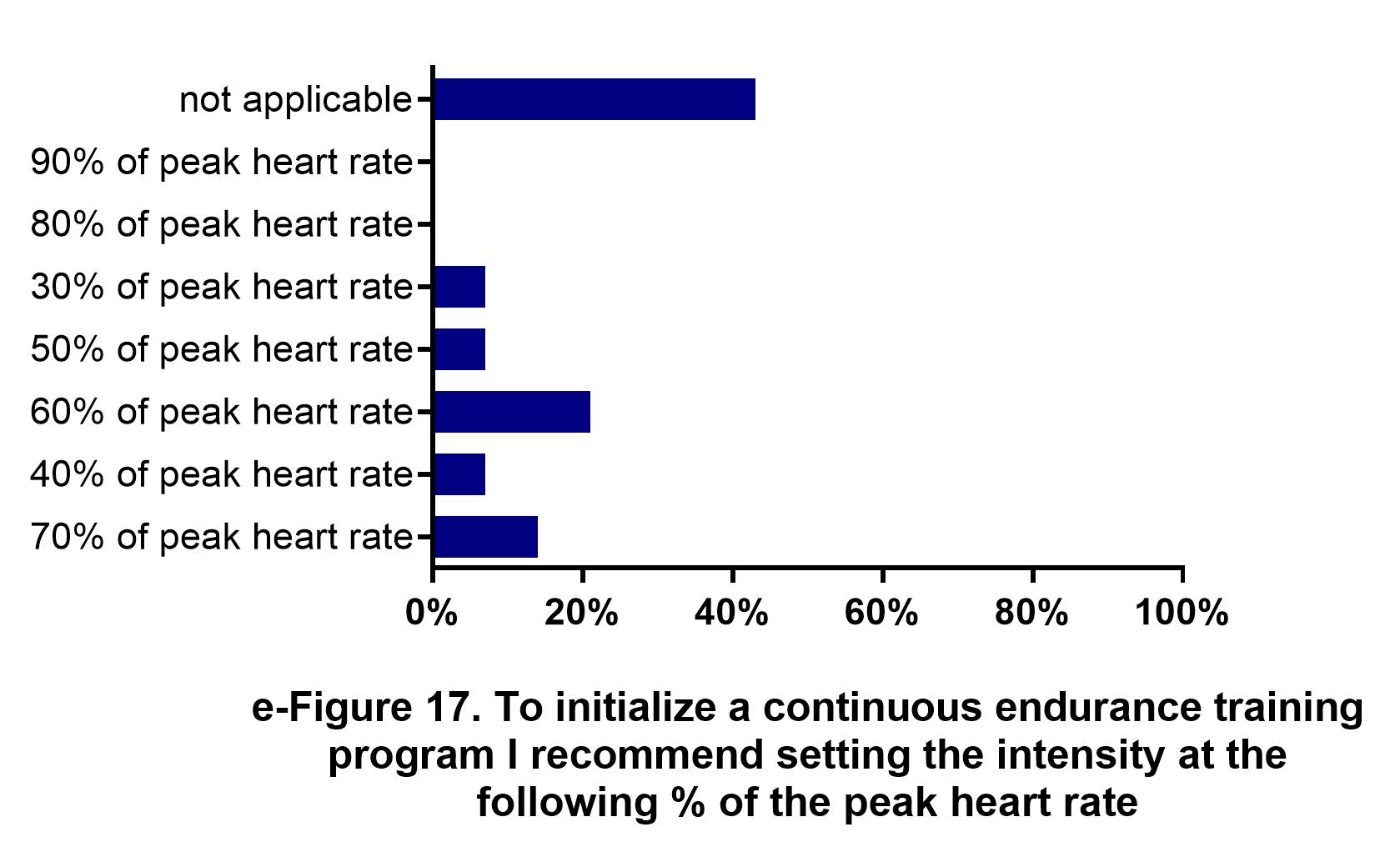

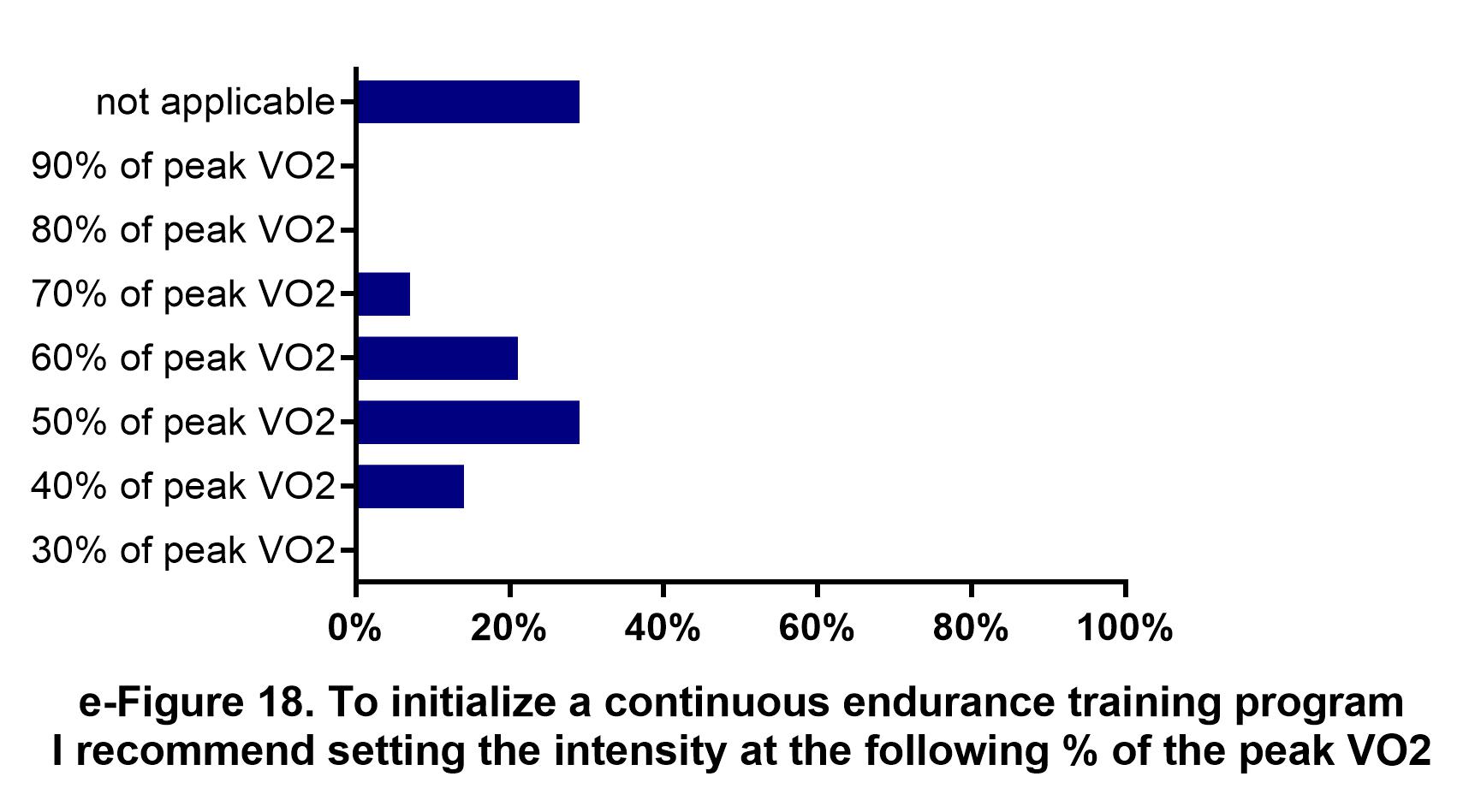

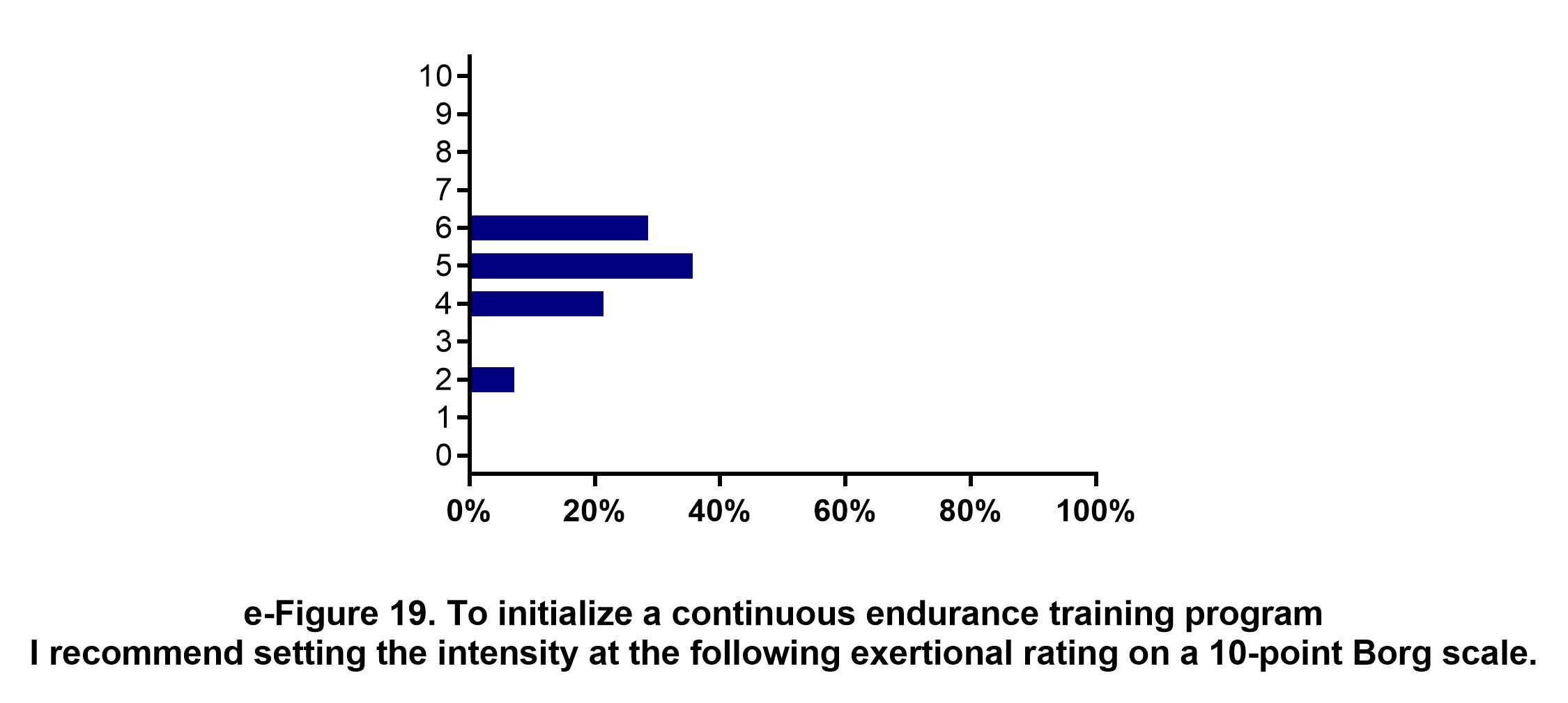

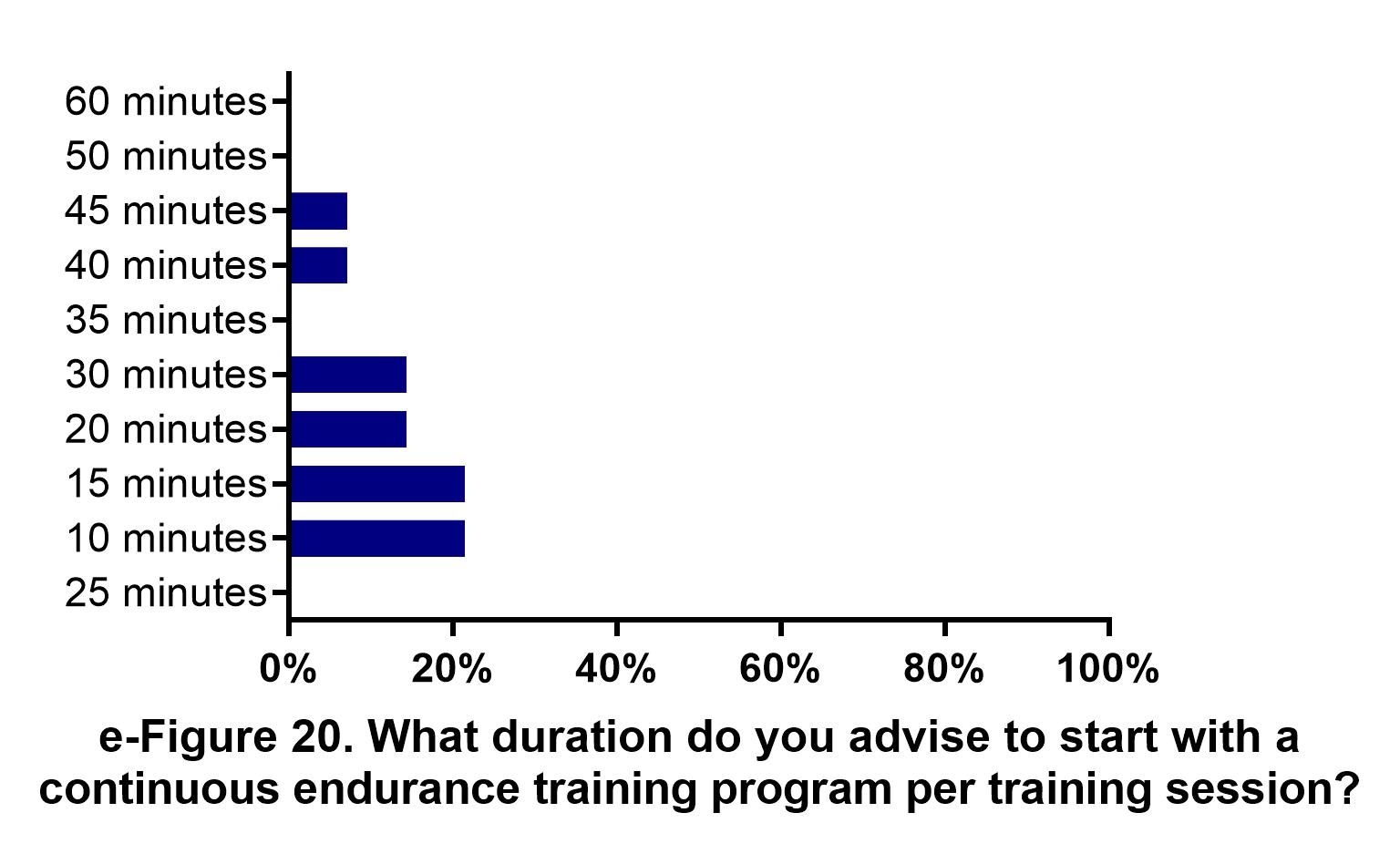

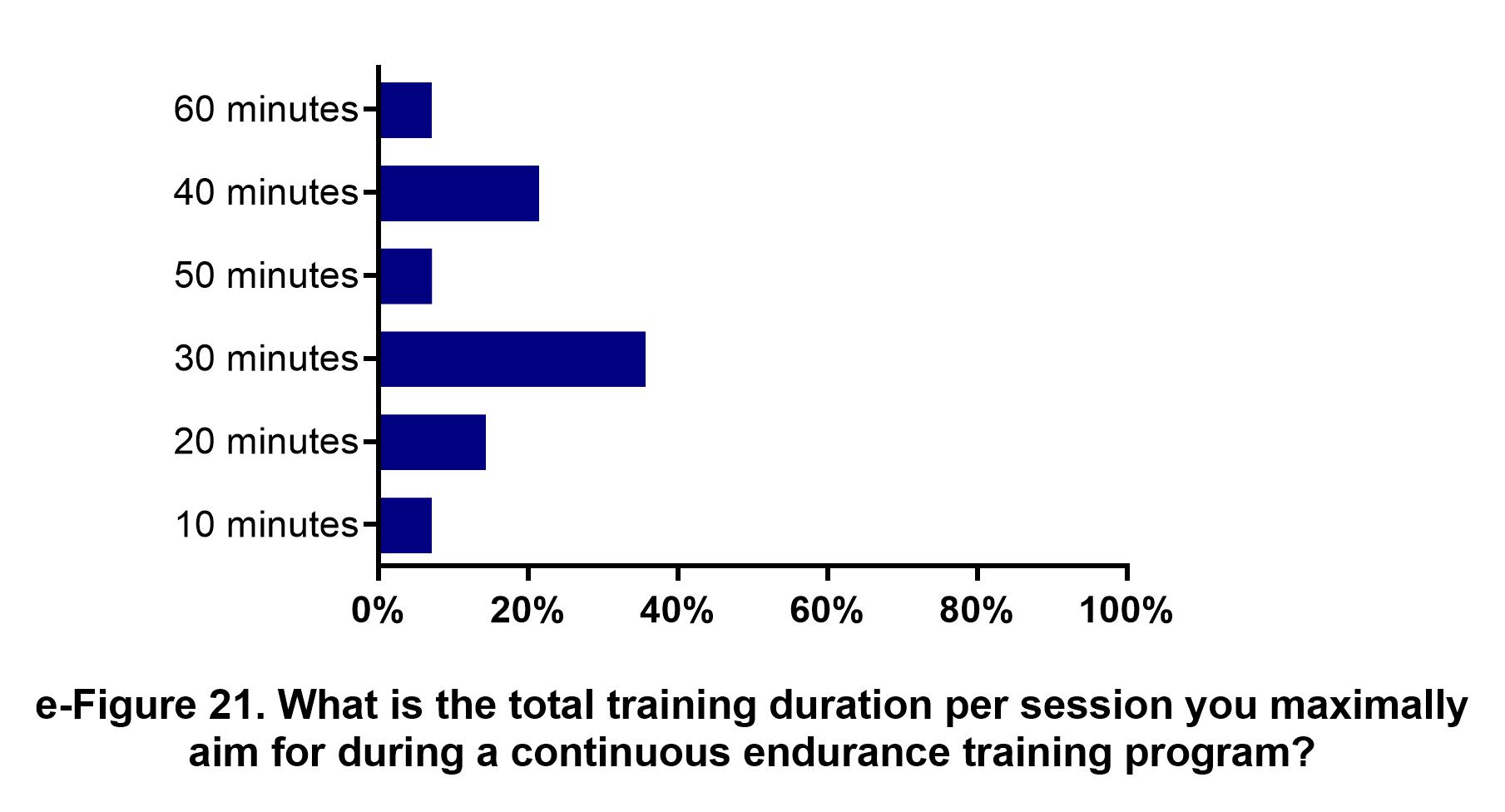

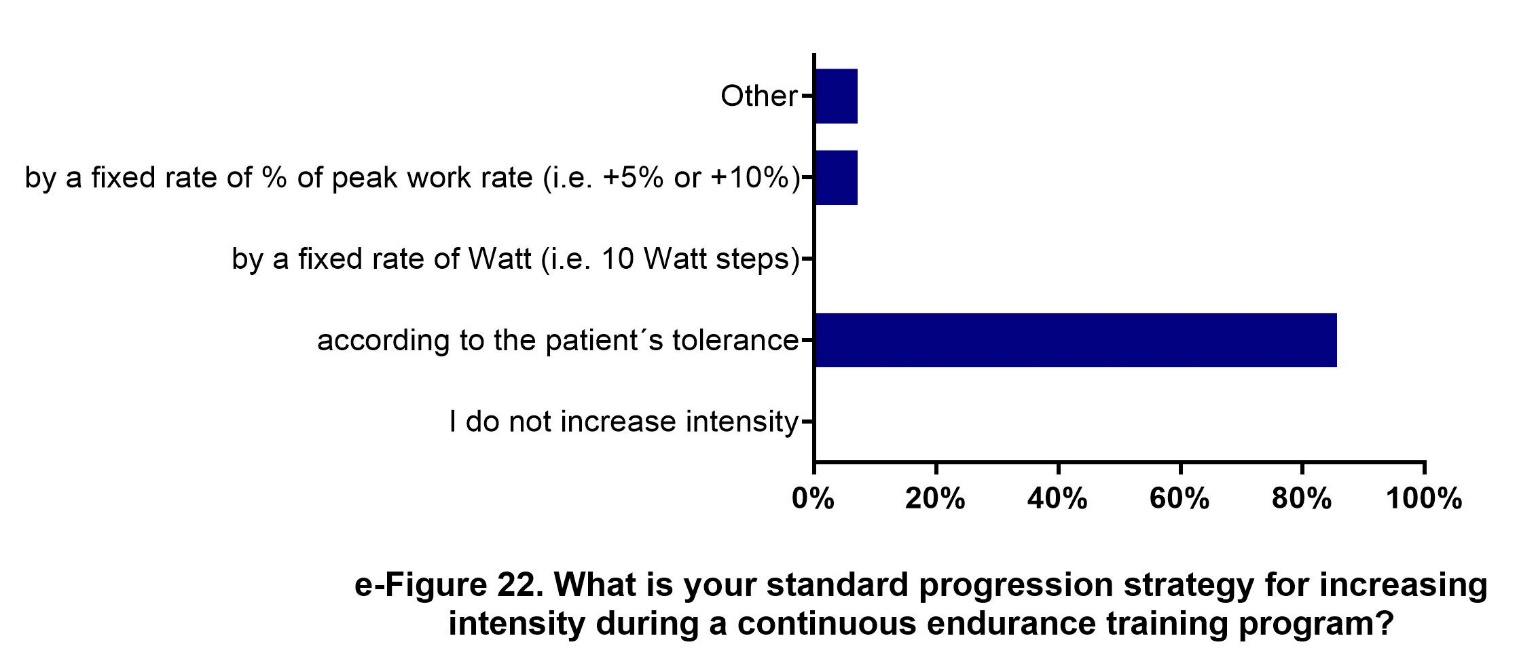

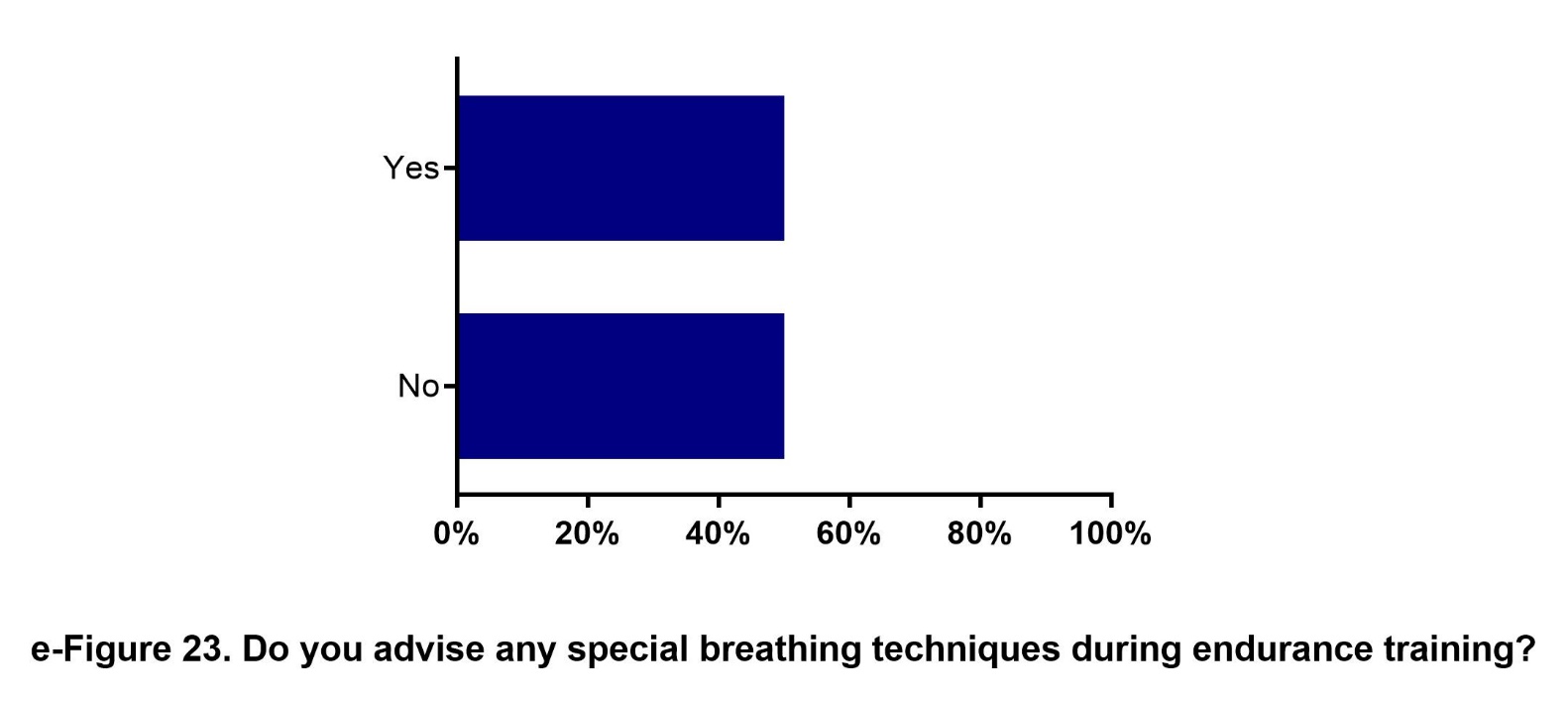

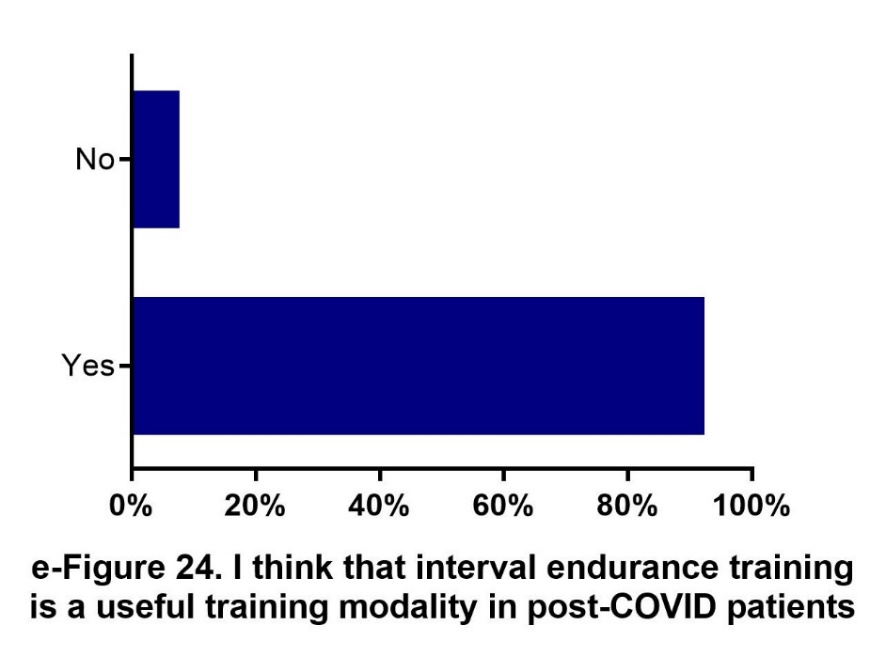

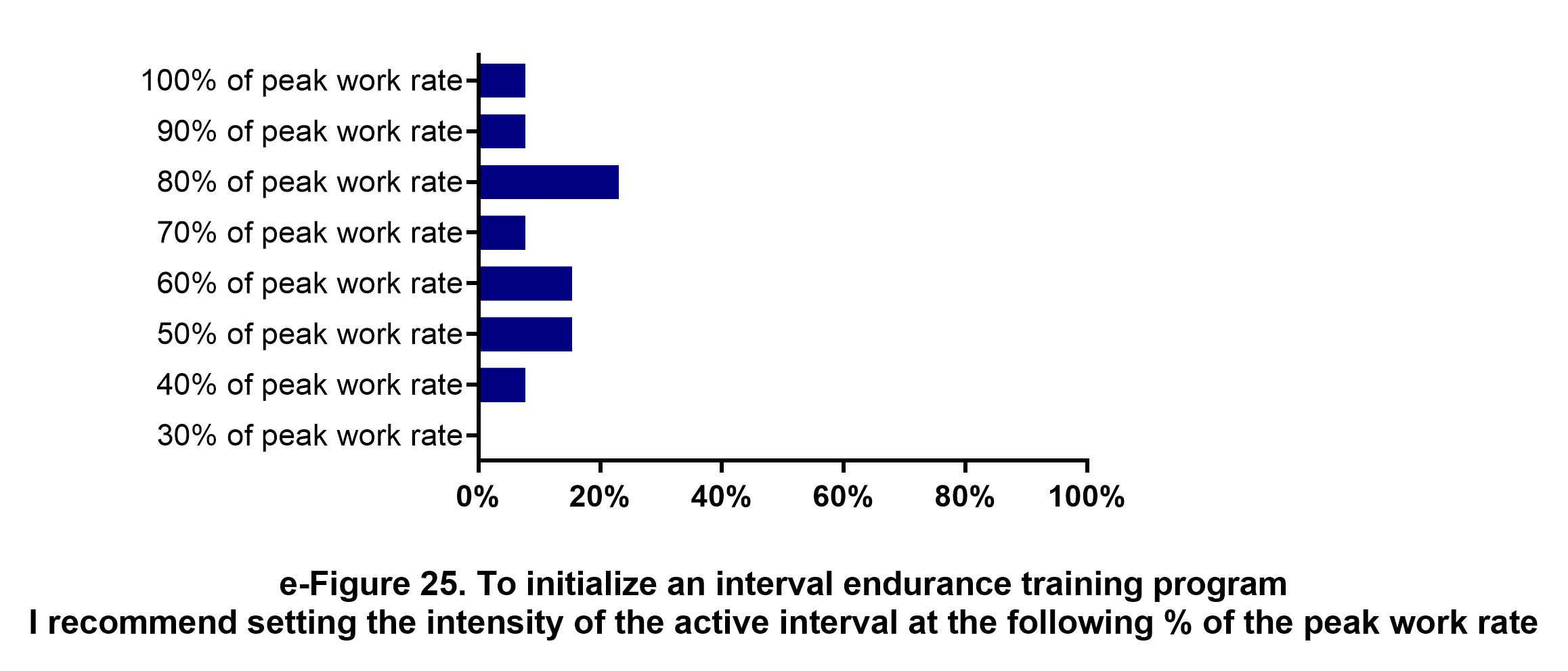

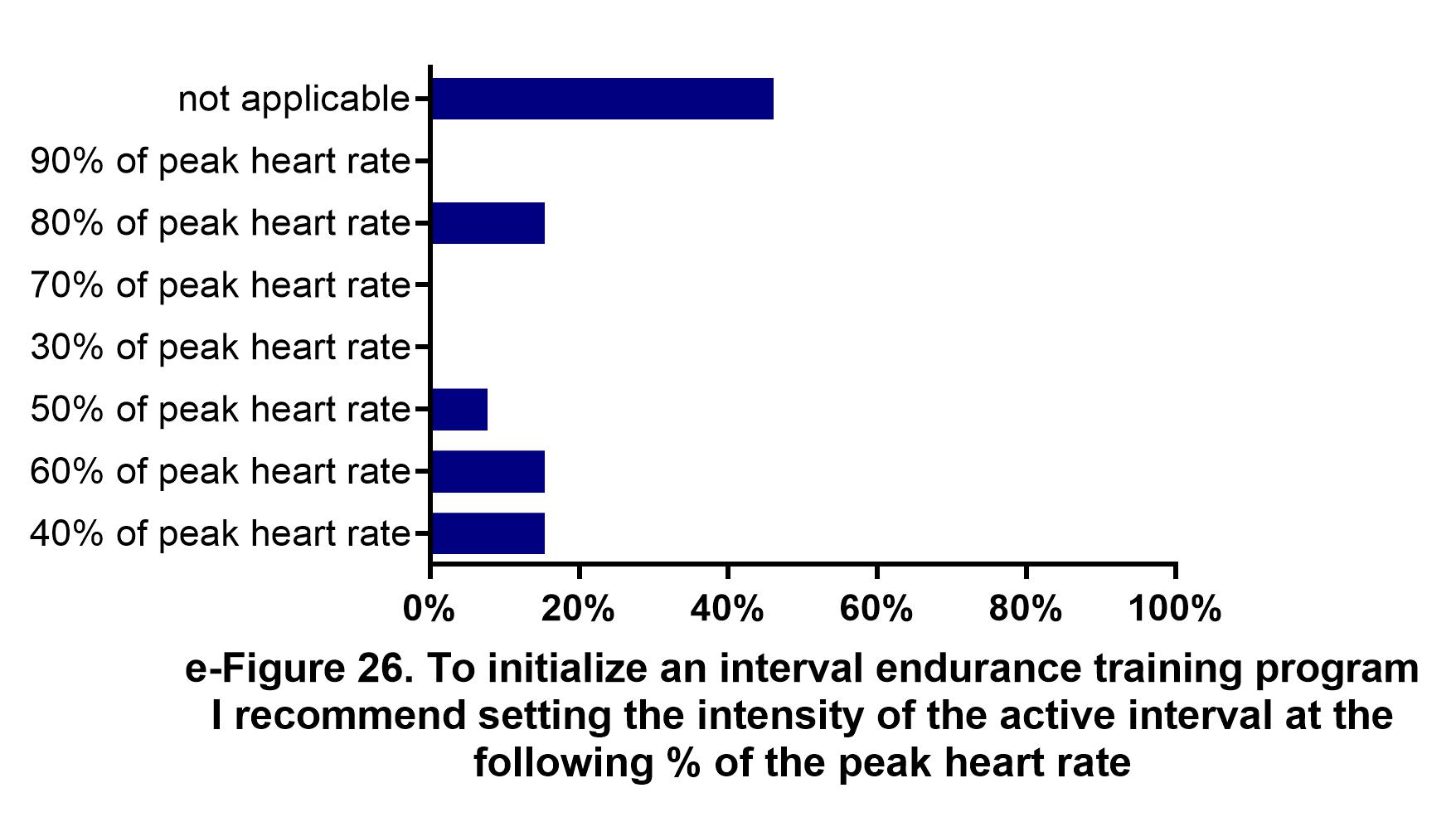

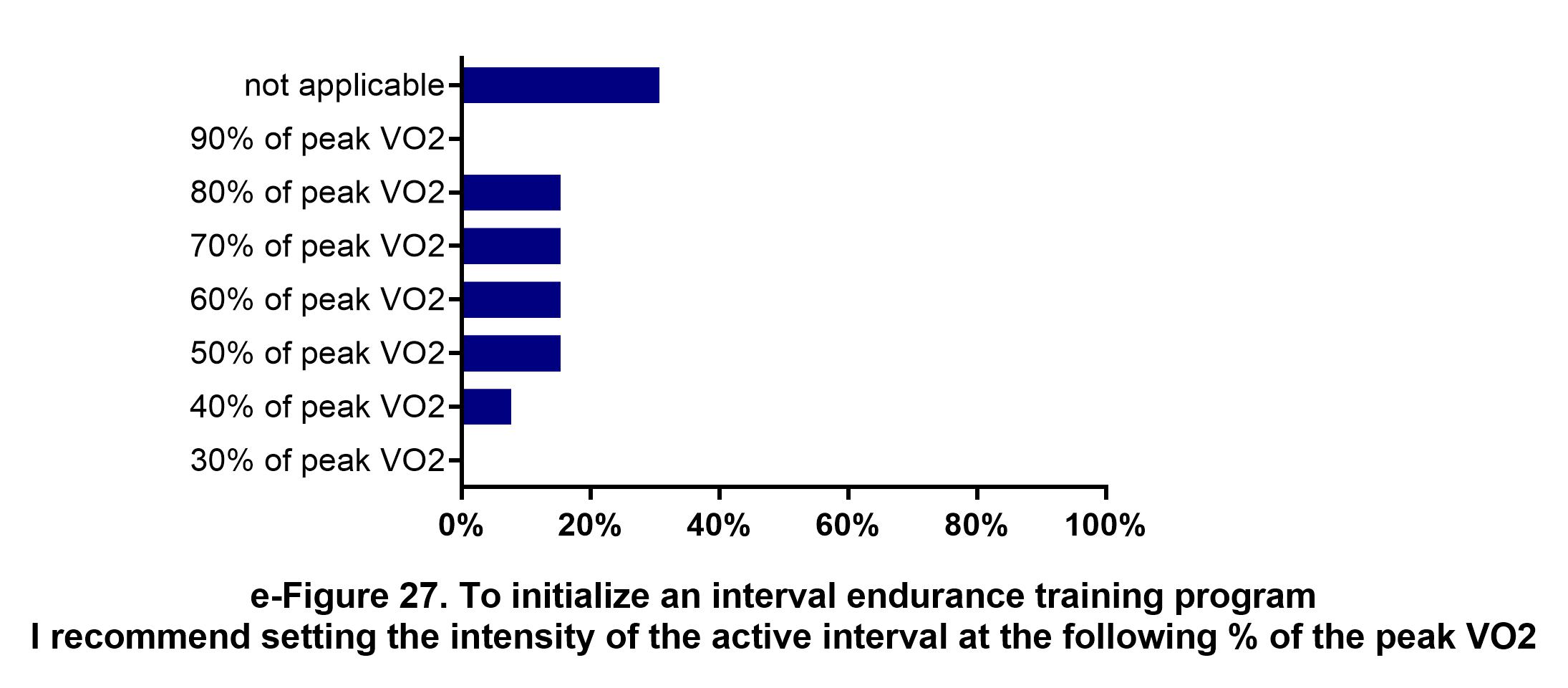

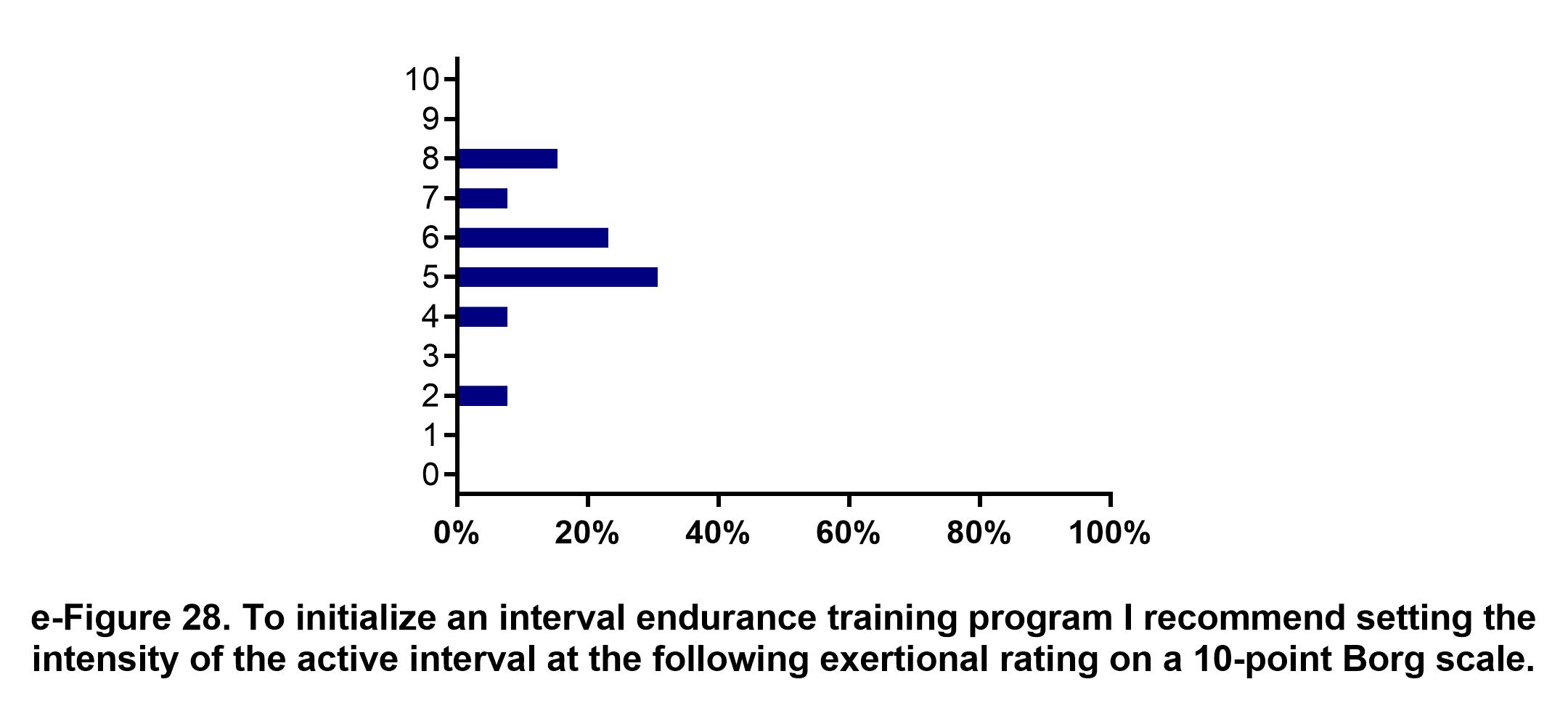

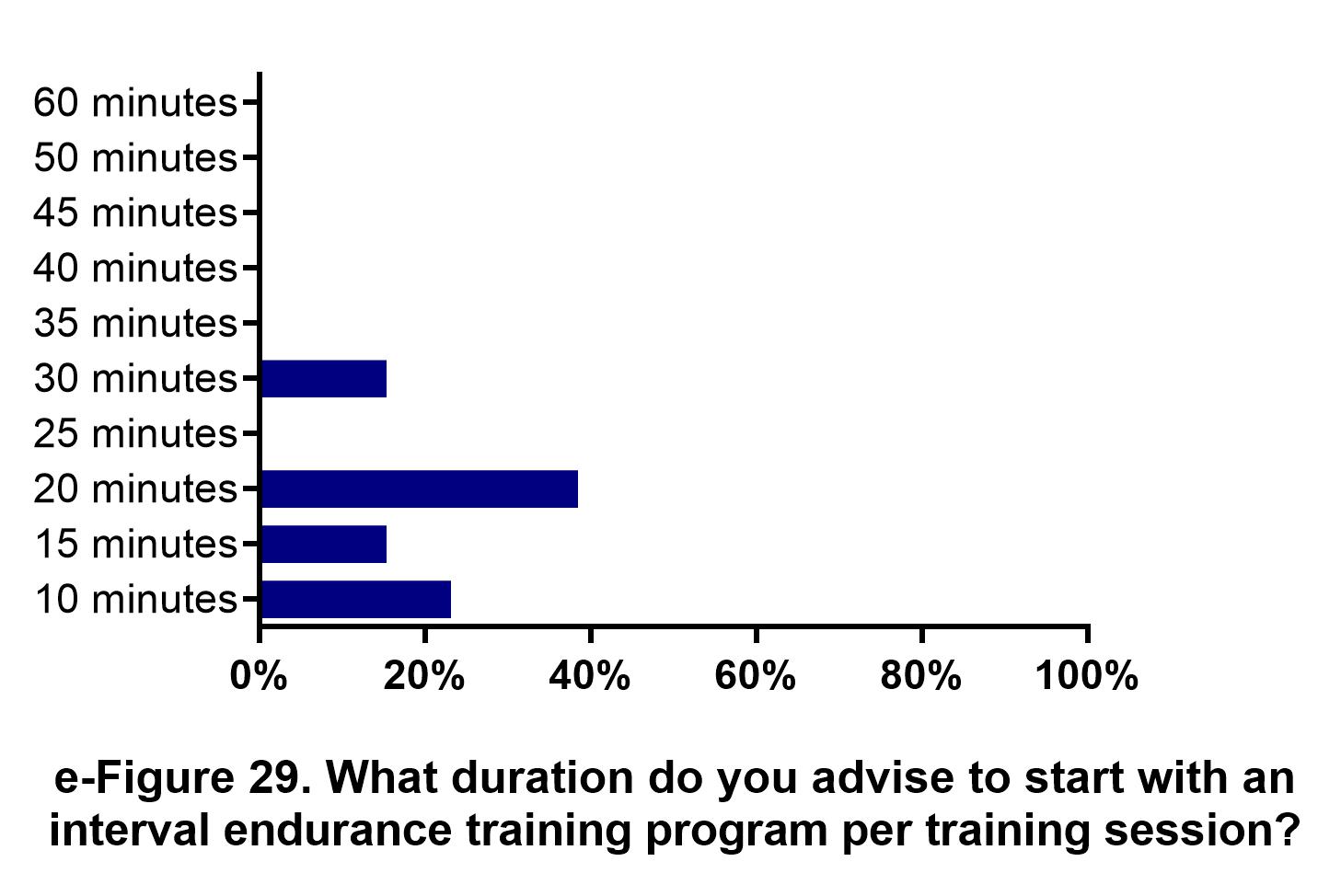

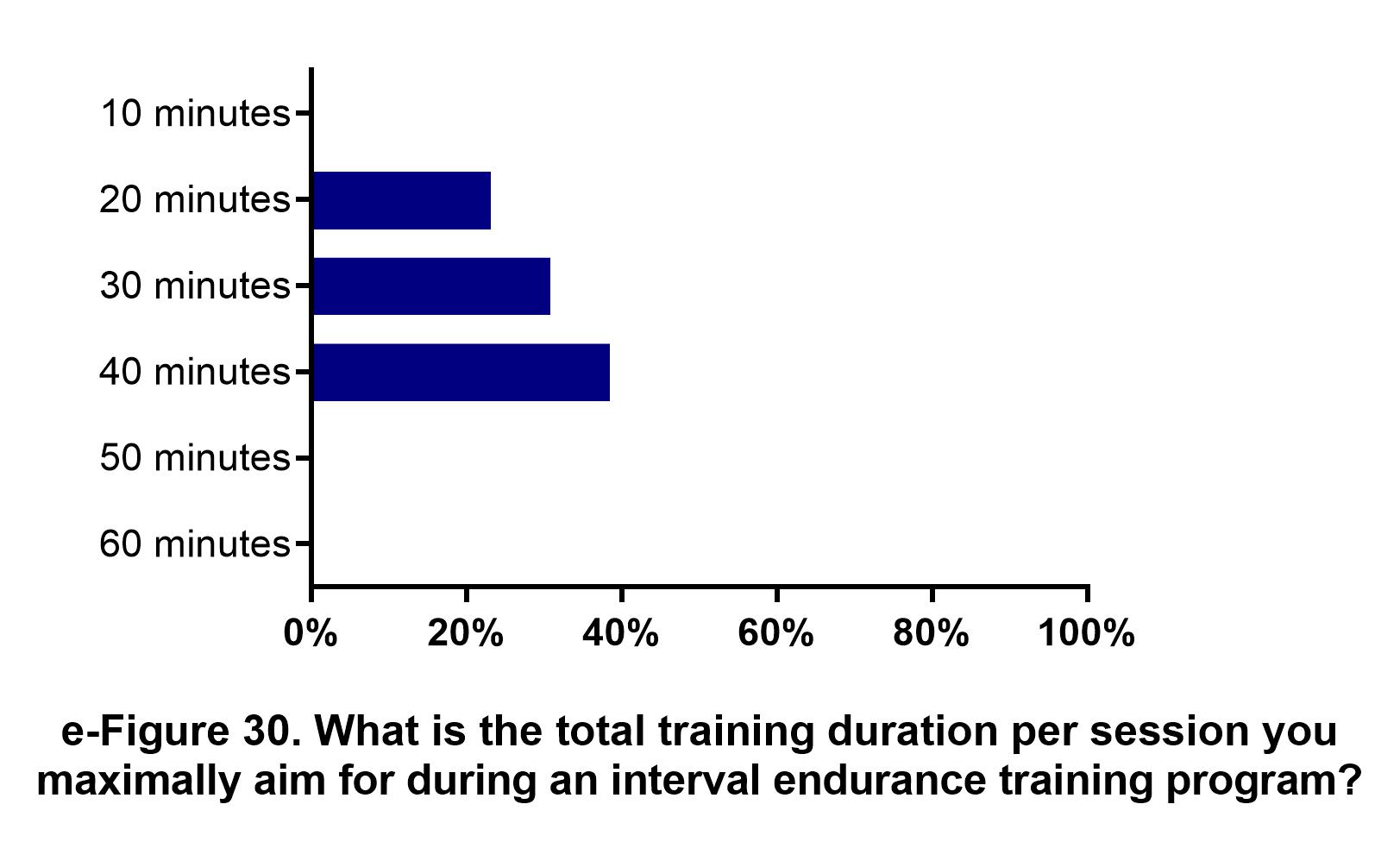

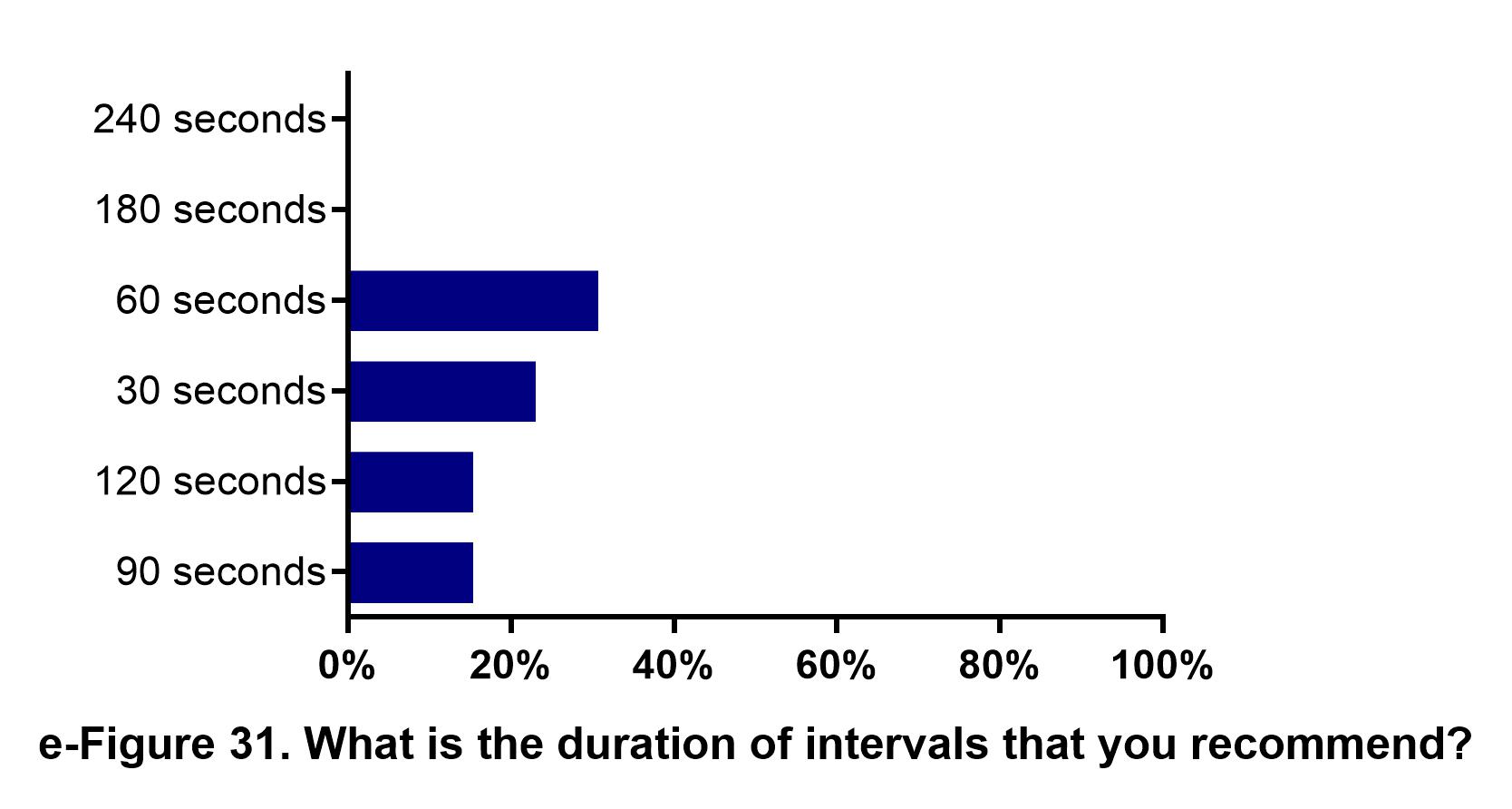

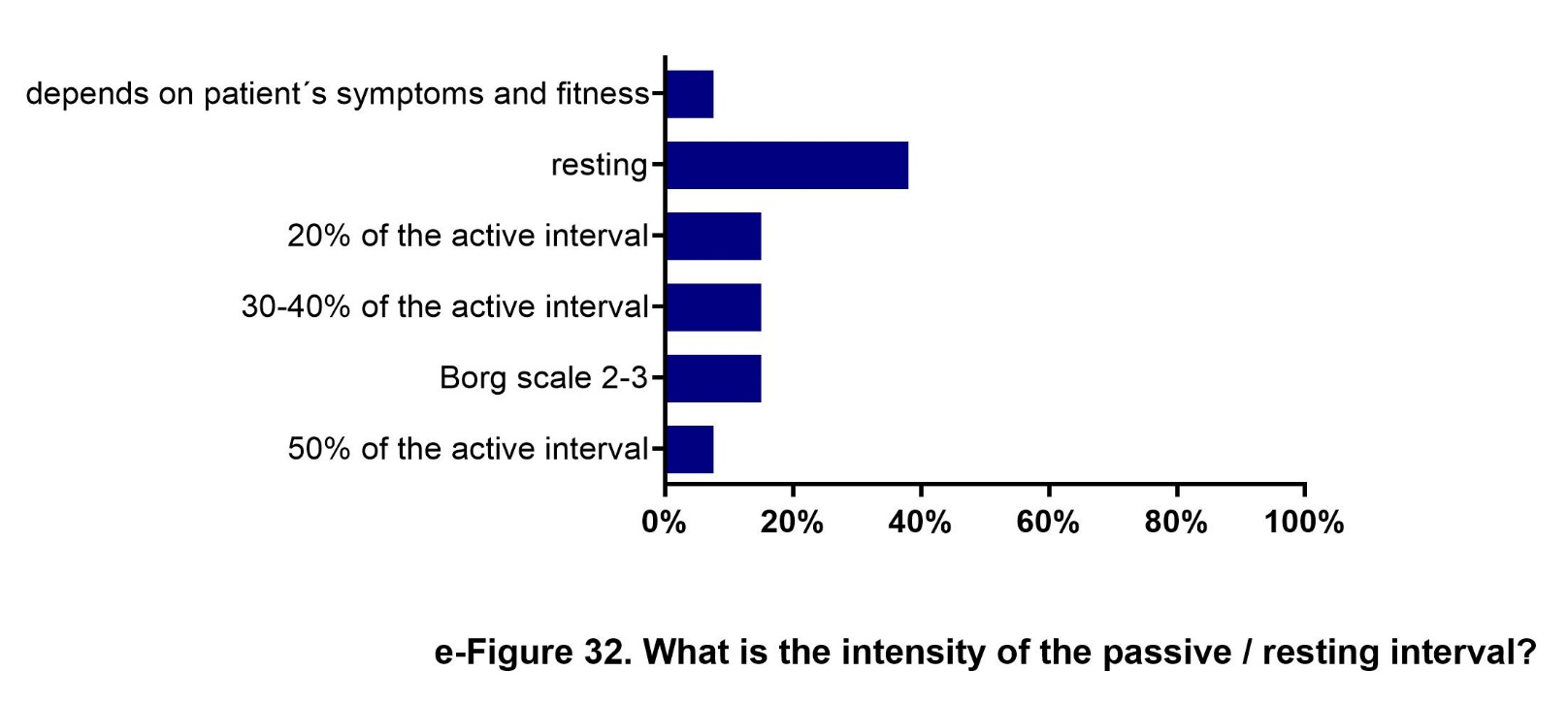

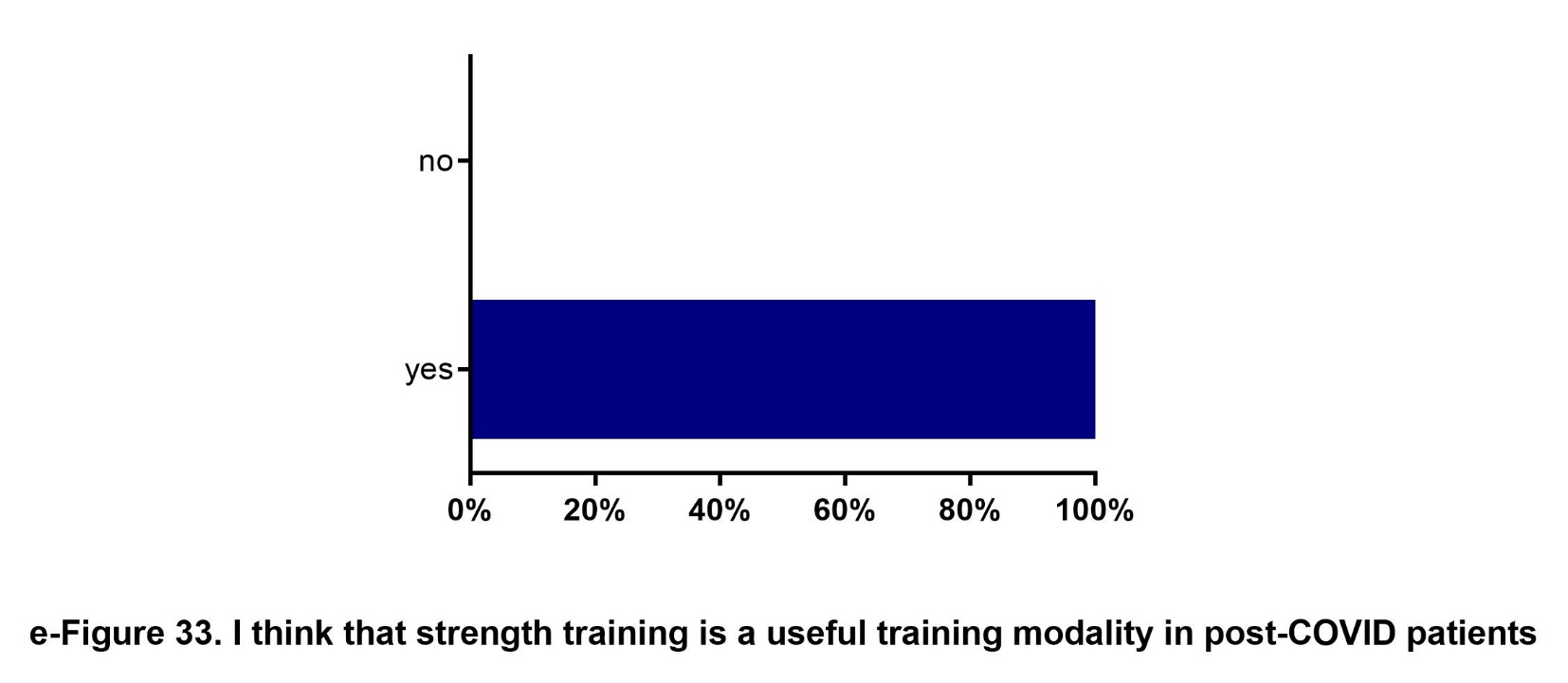

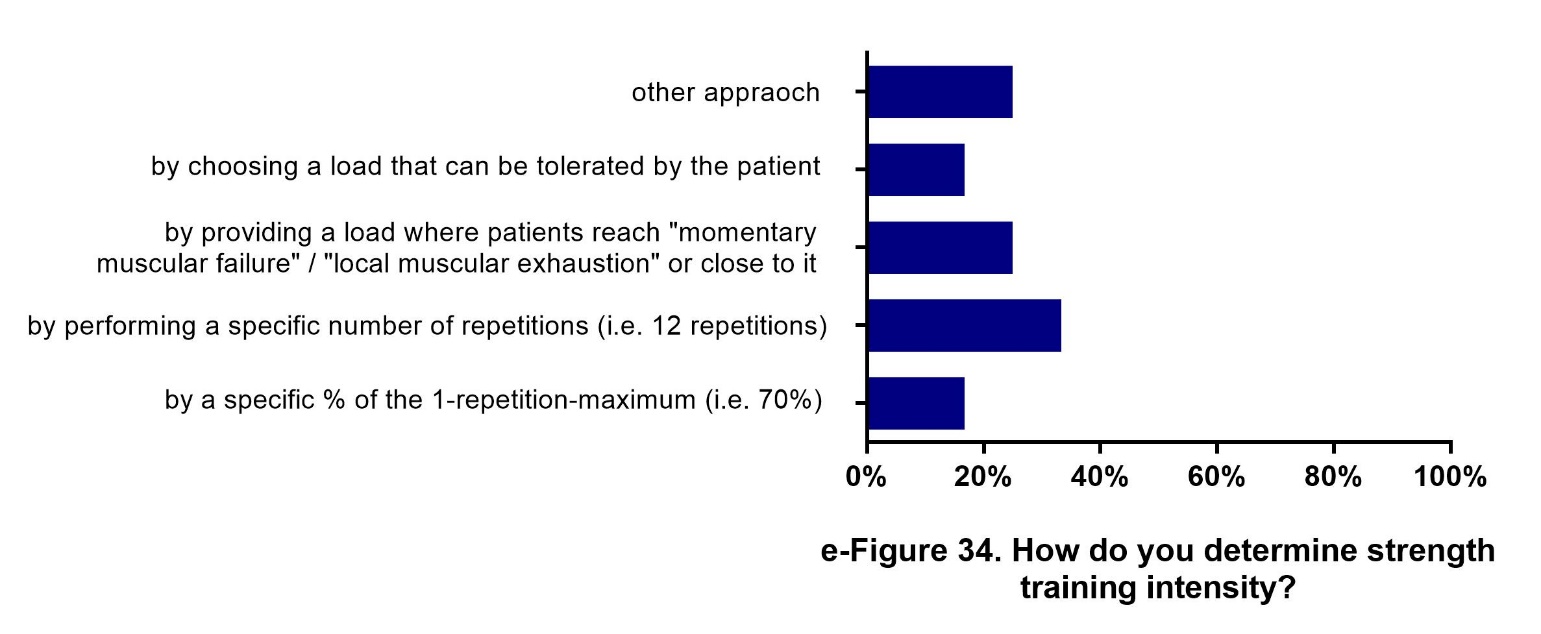

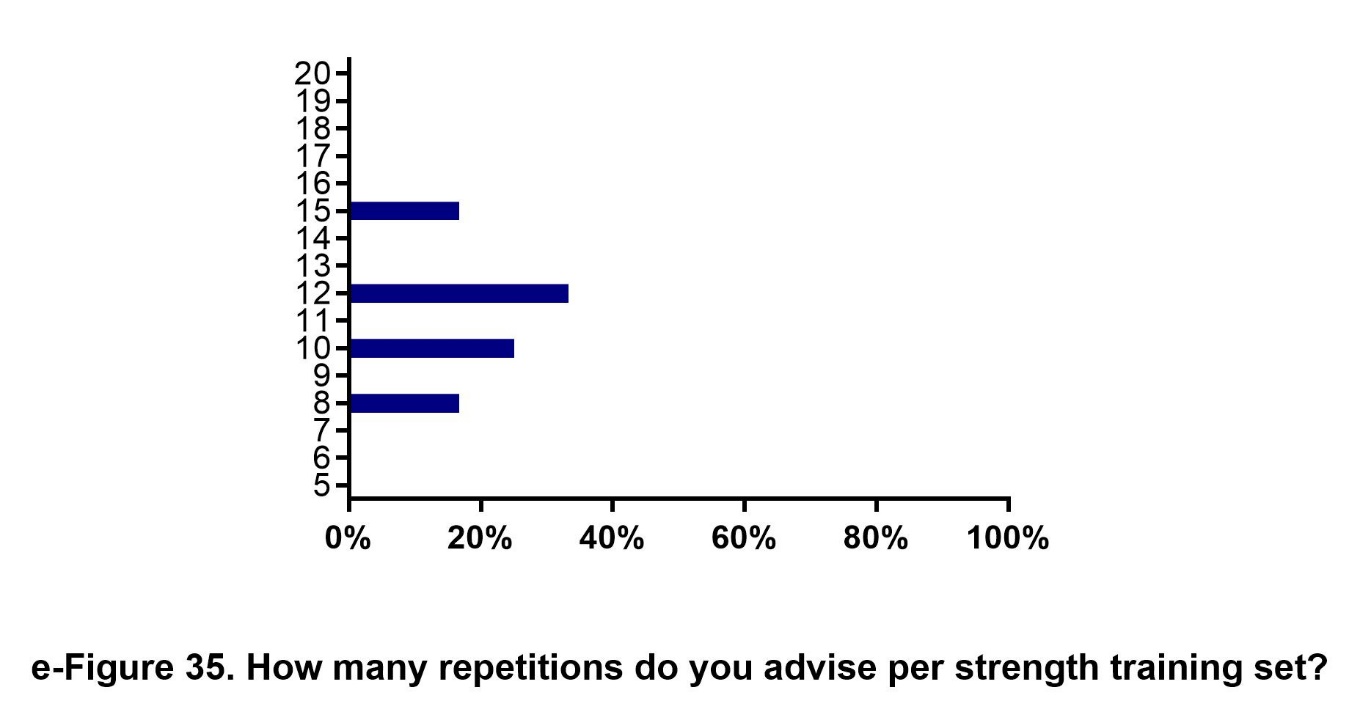

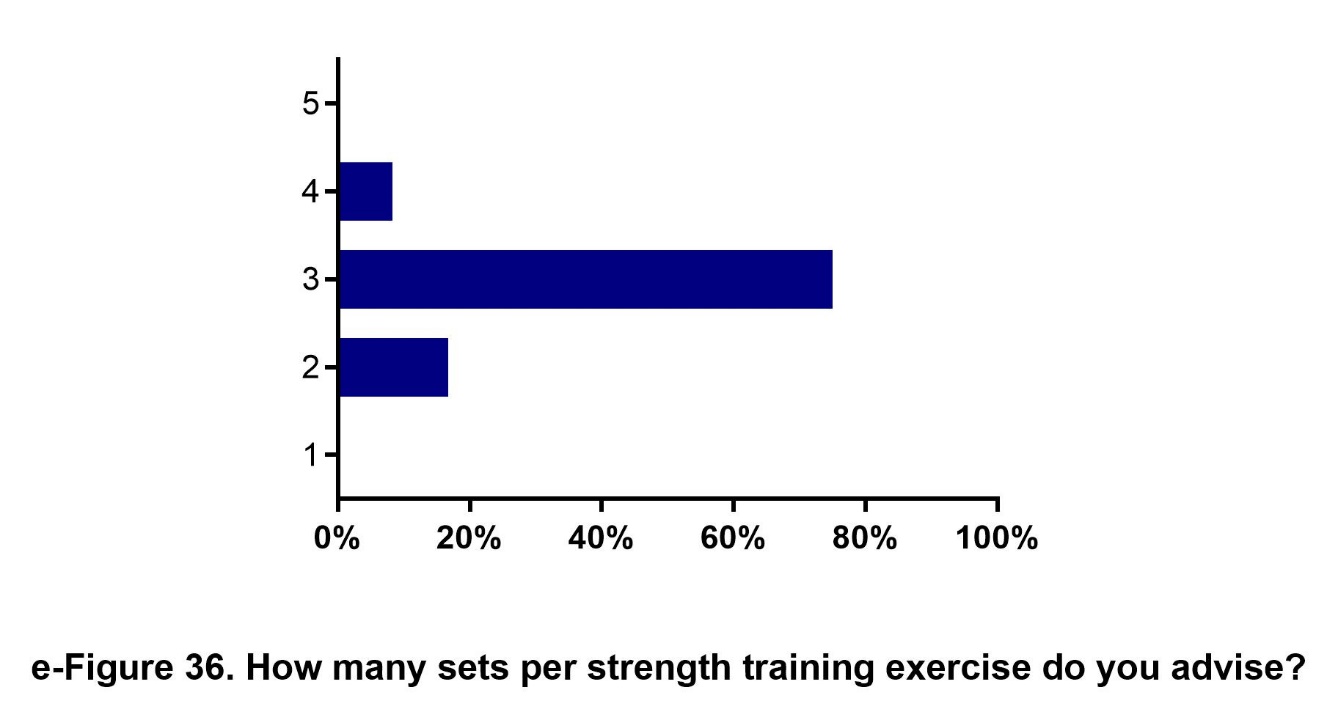

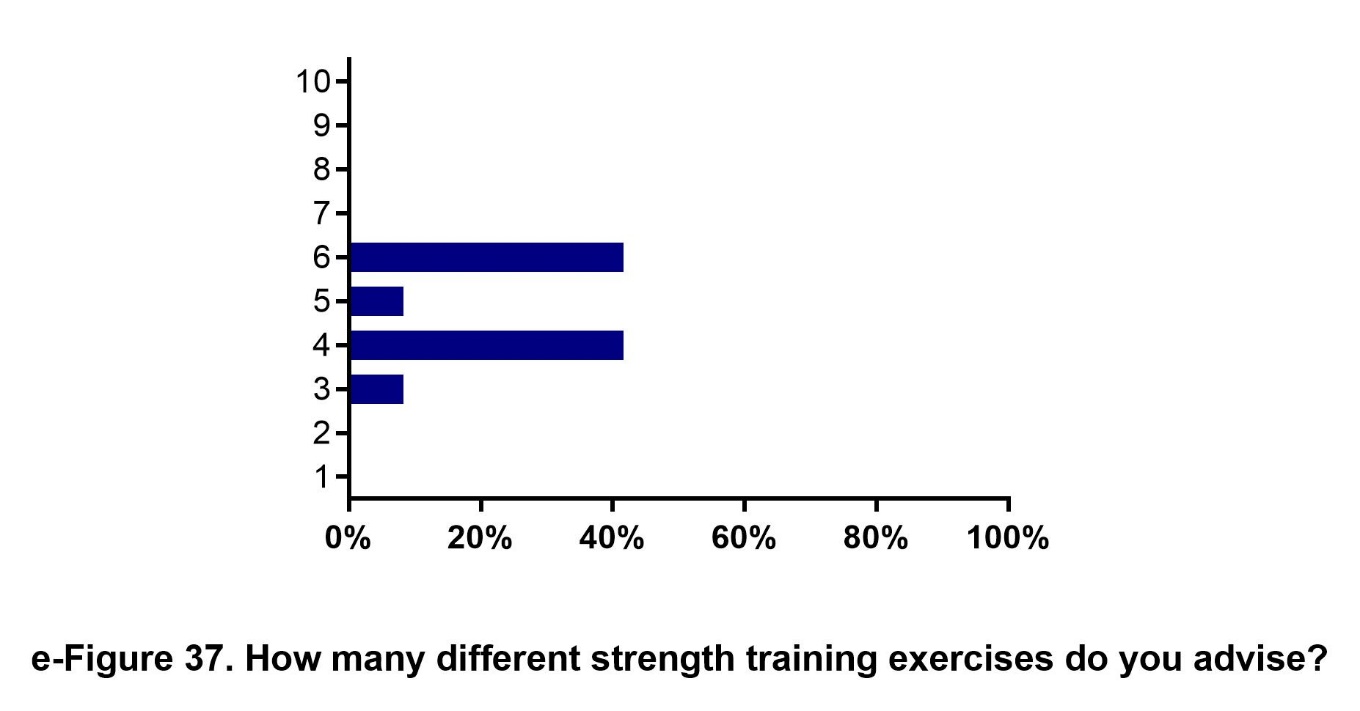

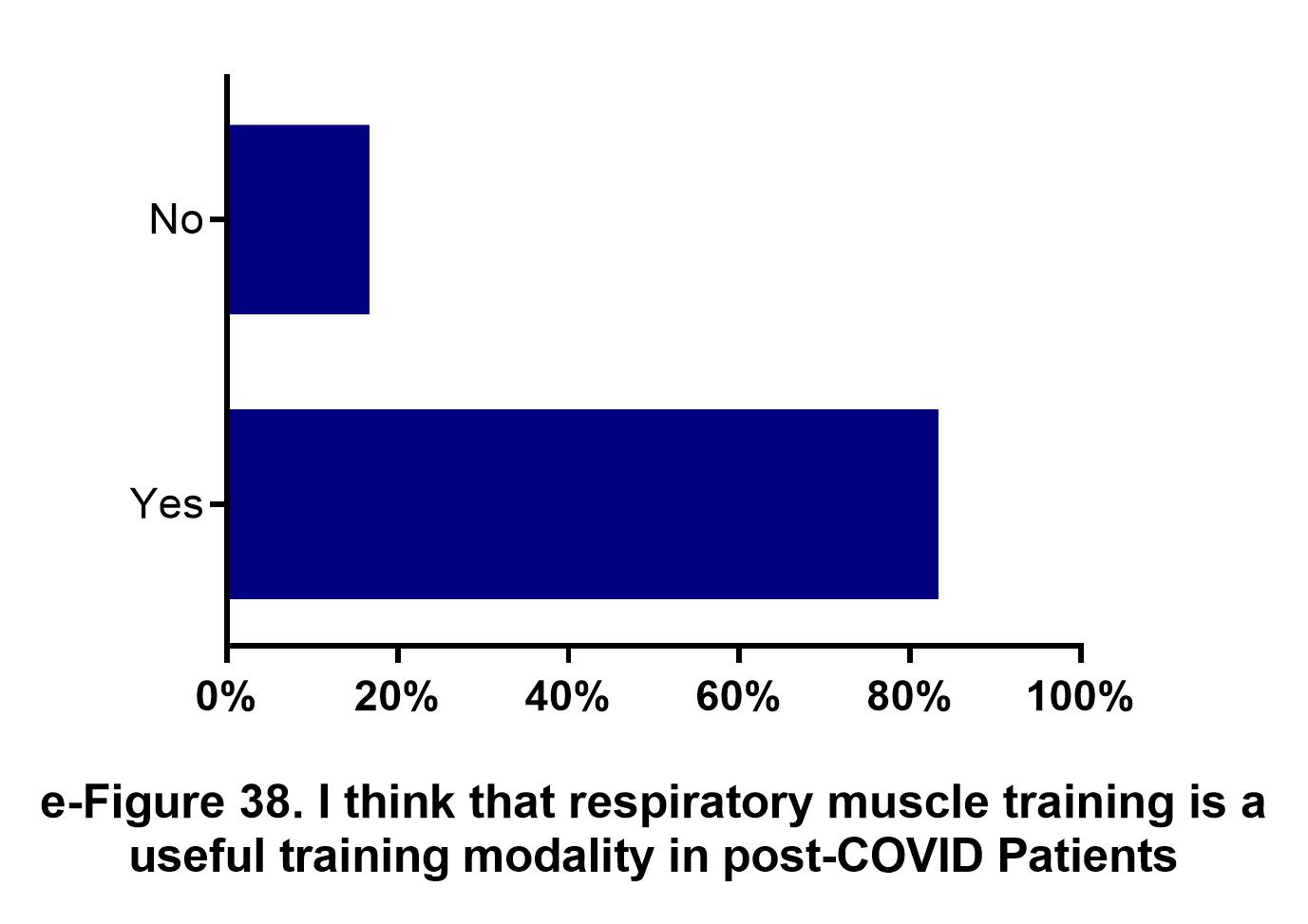

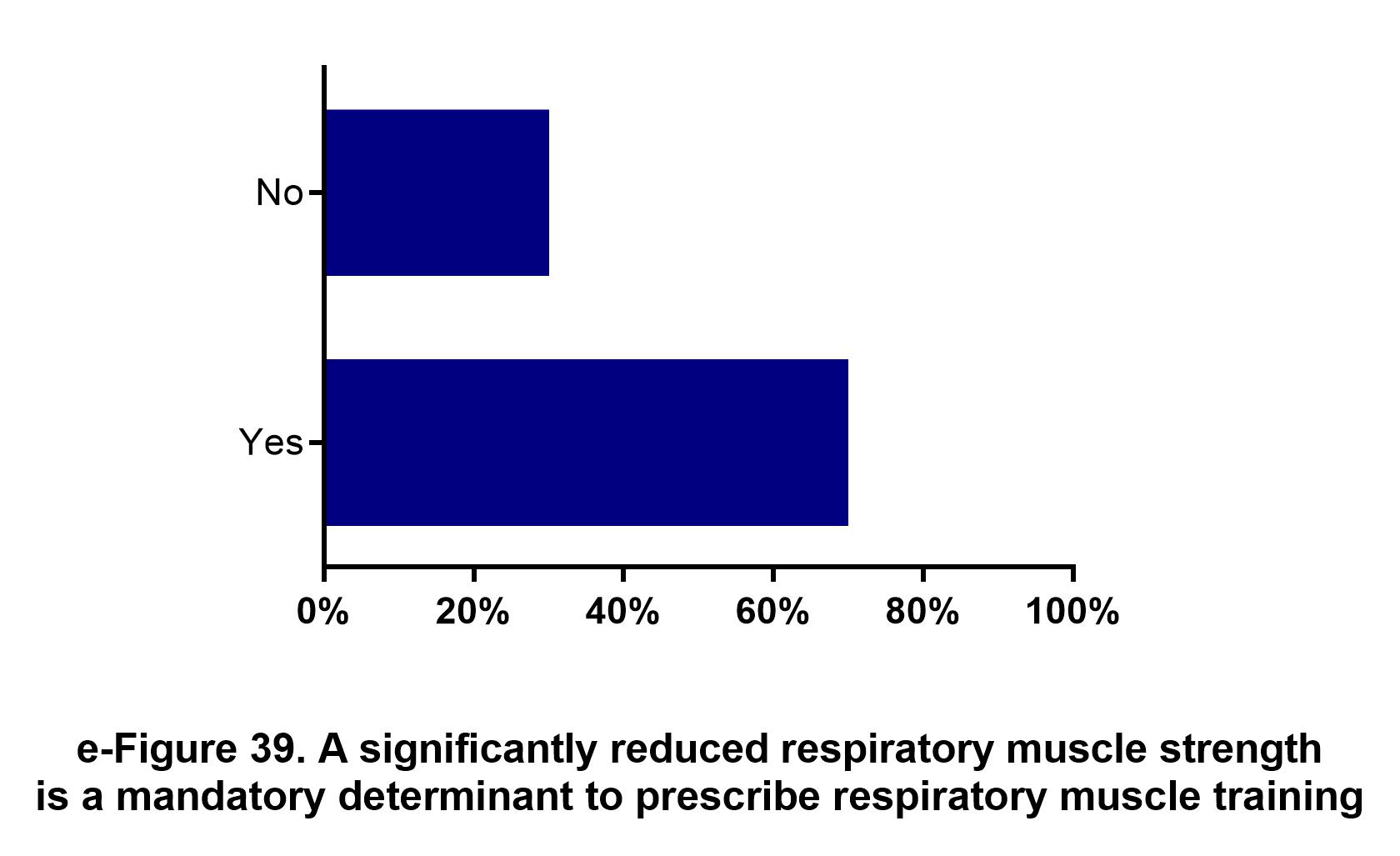

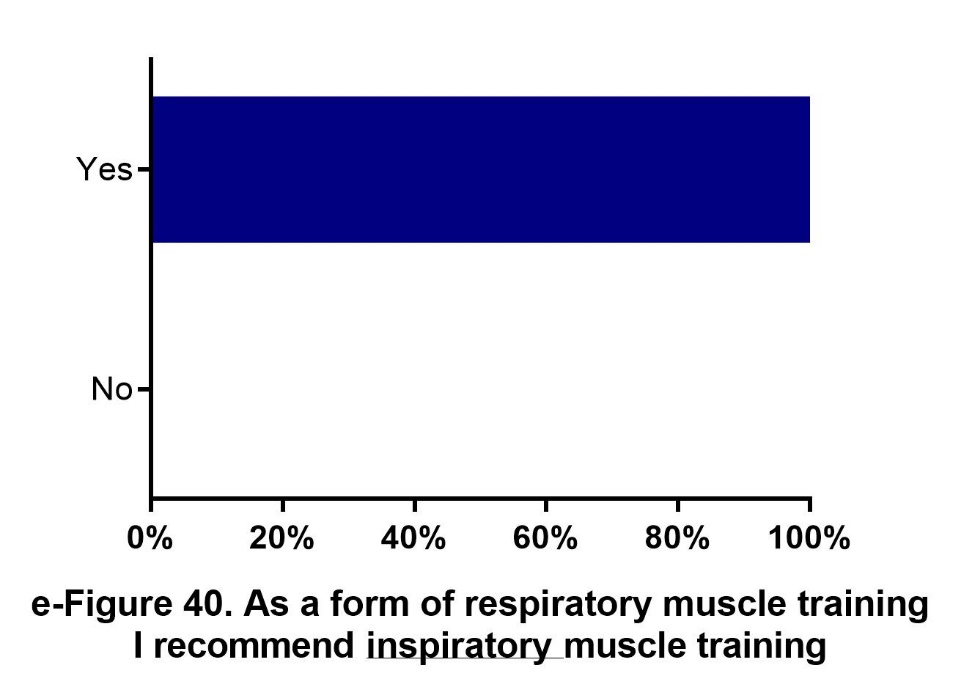

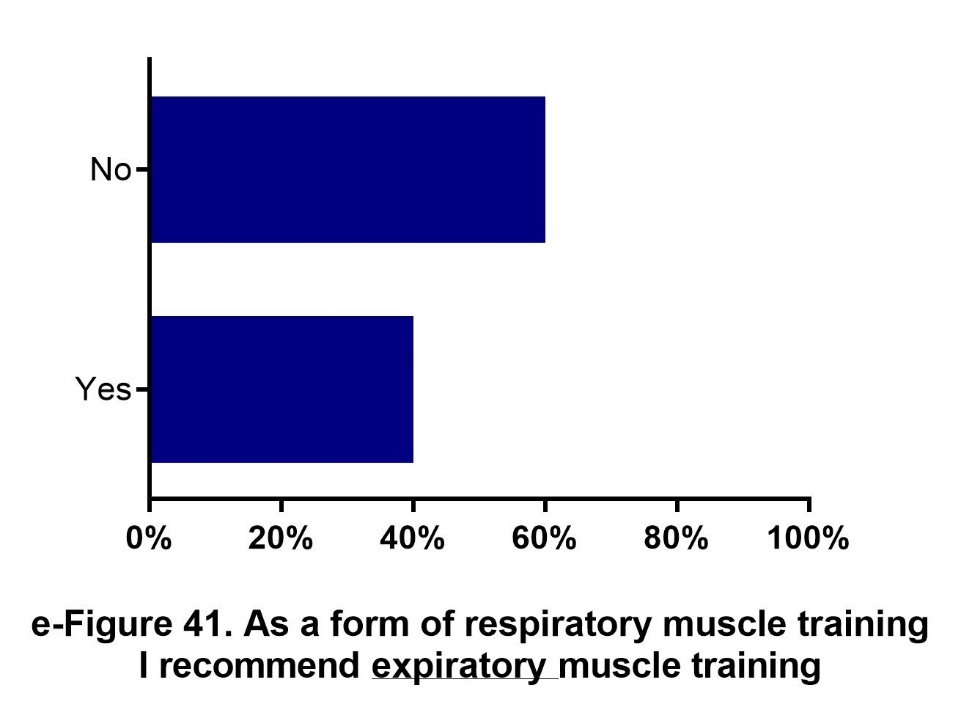

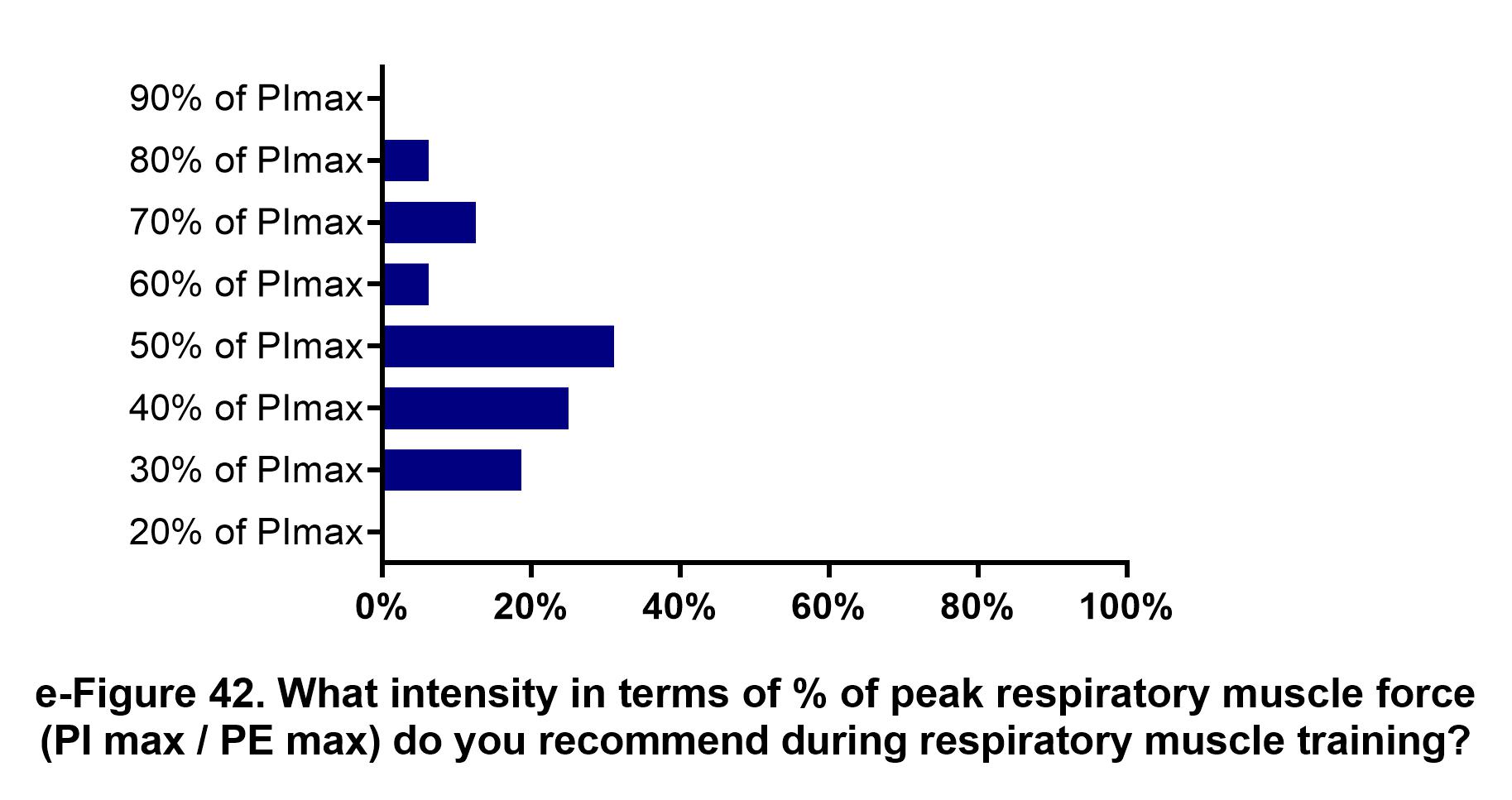

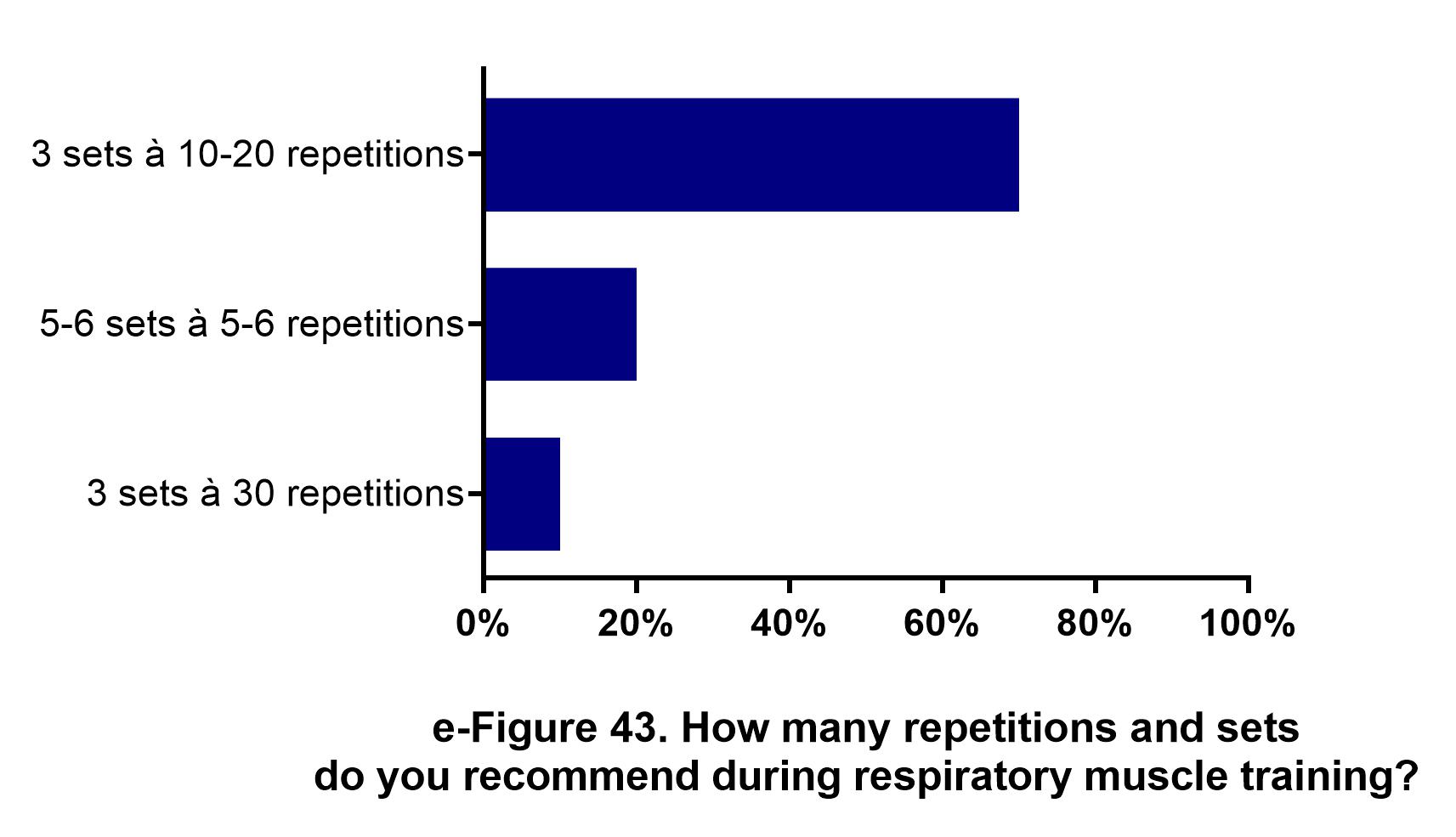

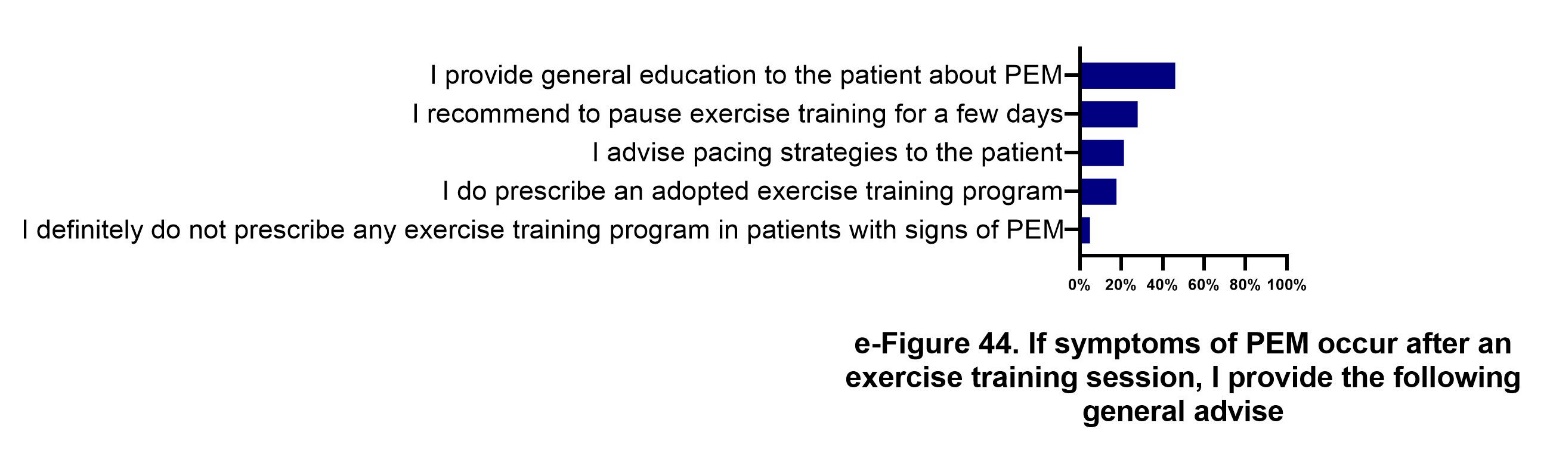

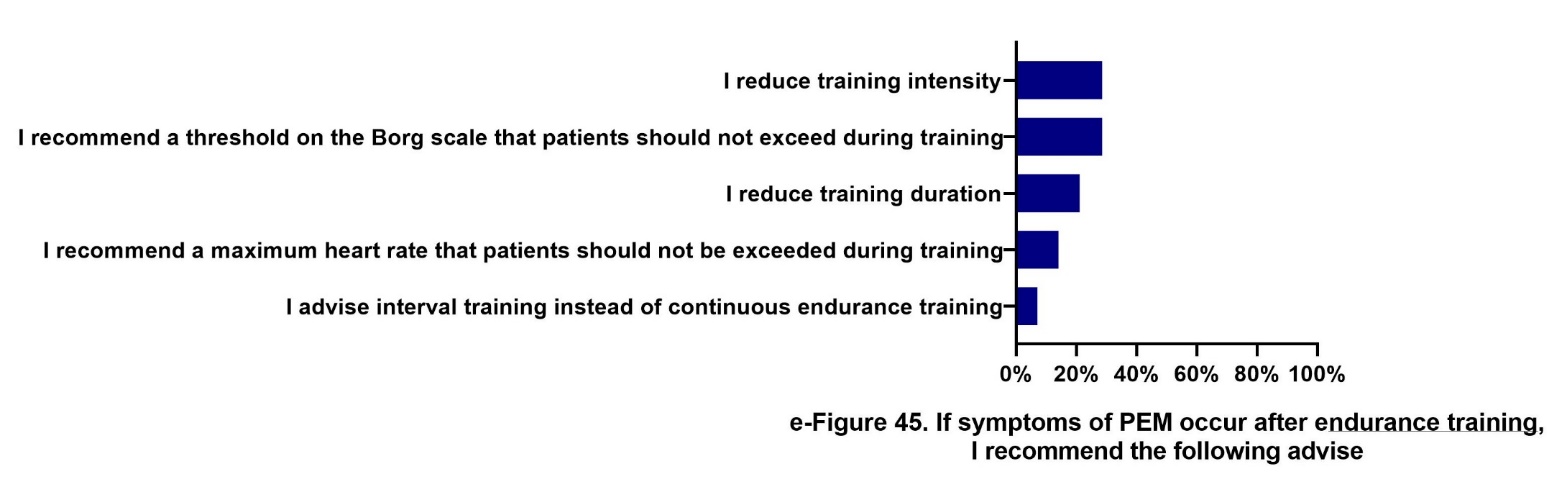

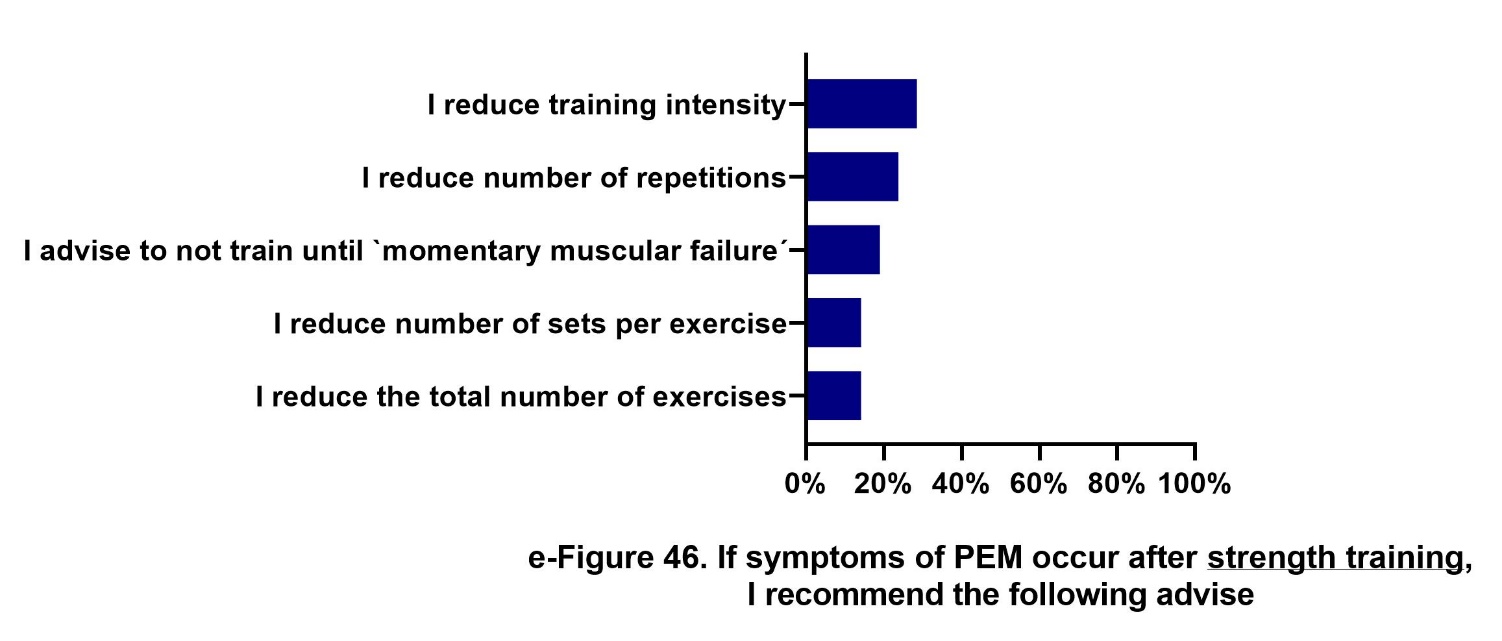


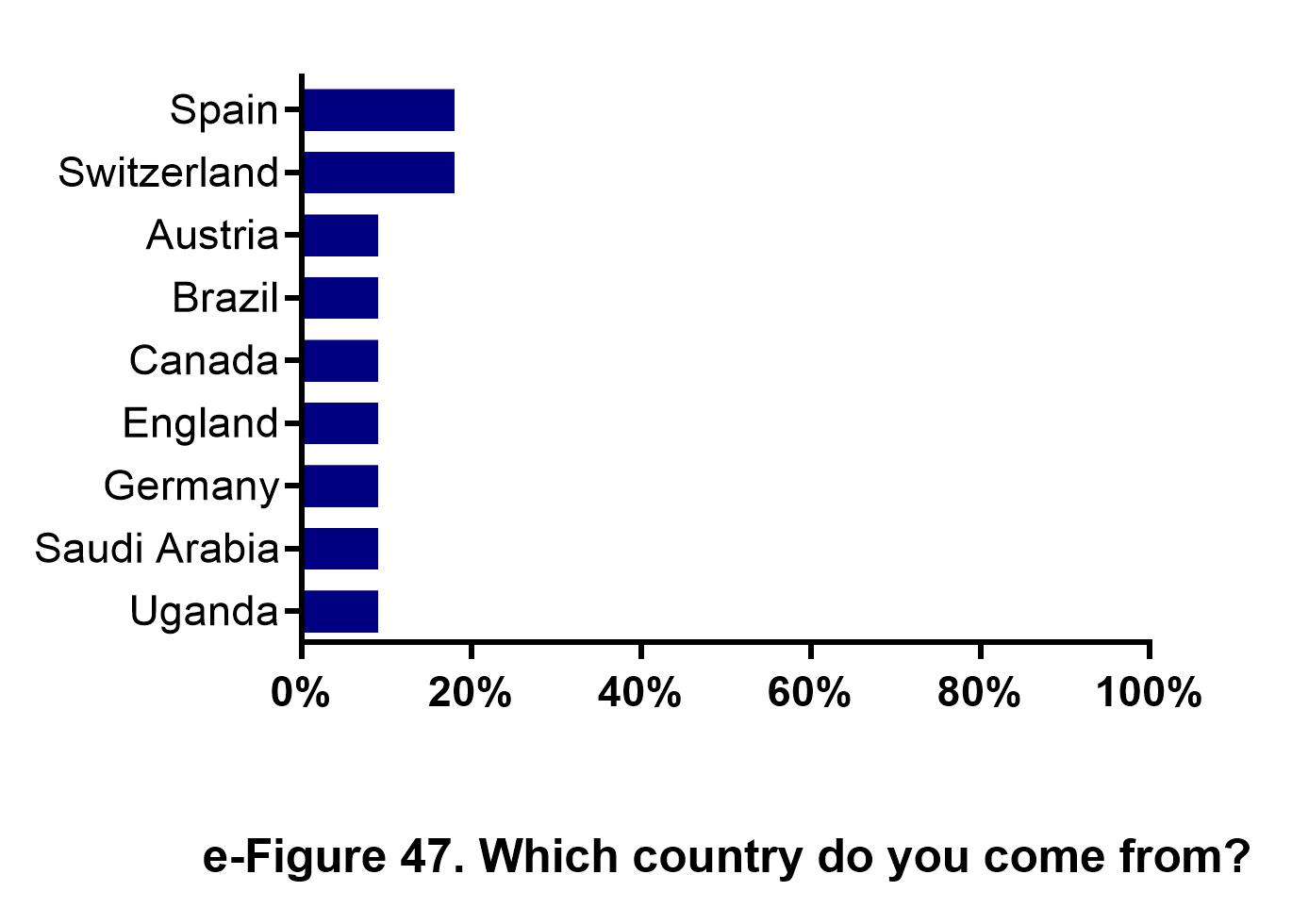

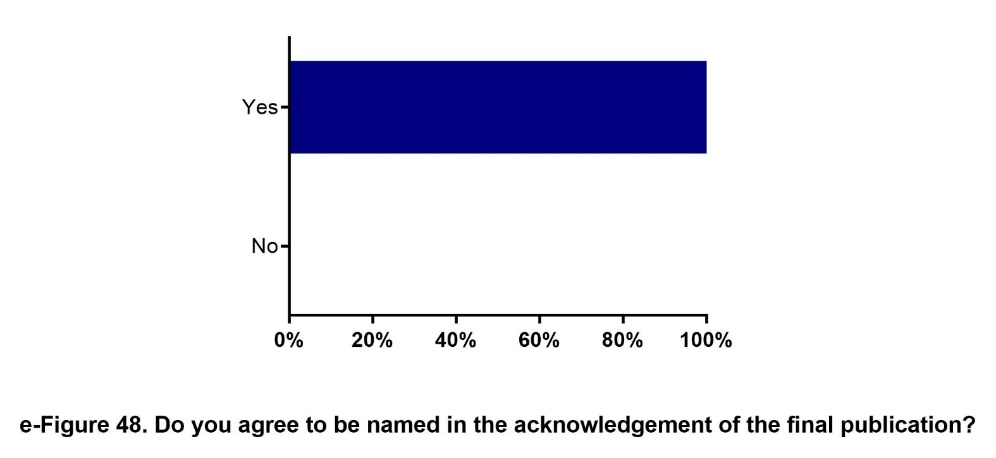

Supplement: Supplementary file 1 — Additional file 1. Supplementary Material Appendix S1. [file 40798_2024_695_MOESM1_ESM.docx]
